# Extracorporeal treatment for ethylene glycol poisoning: systematic review and recommendations from the EXTRIP workgroup

**DOI:** 10.1186/s13054-022-04227-2

**Published:** 2023-02-10

**Authors:** Marc Ghannoum, Sophie Gosselin, Robert S. Hoffman, Valery Lavergne, Bruno Mégarbane, Hossein Hassanian-Moghaddam, Maria Rif, Siba Kallab, Steven Bird, David M. Wood, Darren M. Roberts, for the EXTRIP Workgroup, Badria Alhatali, Kurt Anseeuw, Ingrid Berling, Josée Bouchard, Timothy E. Bunchman, Diane P. Calello, Paul K. Chin, Kent Doi, Tais Galvao, David S. Goldfarb, Lotte C. G. Hoegberg, Sofia Kebede, Jan T. Kielstein, Andrew Lewington, Yi Li, Etienne M. Macedo, Rob MacLaren, James B. Mowry, Thomas D. Nolin, Marlies Ostermann, Ai Peng, Jean-Philippe Roy, Greene Shepherd, Anitha Vijayan, Steven J. Walsh, Anselm Wong, Christopher Yates

**Affiliations:** 1 Research Center, CIUSSS du Nord-de-l’île-de-Montréal, University of Montreal, Montreal, QC Canada; 2 Nephrology Division, NYU Langone Health, NYU Grossman School of Medicine, New York, NY USA; 3 Department of Nephrology and Hypertension, University Medical Center Utrecht, Utrecht University, Utrecht, The Netherlands; 4 Centre Intégré de Santé et de Services Sociaux (CISSS) de la Montérégie-Centre Emergency Department, Hôpital Charles-Lemoyne, Greenfield Park, QC Canada; 5 Faculté de Médecine et Sciences de la Santé, Université de Sherbrooke, Sherbrooke, Canada; 6 Centre Antipoison du Québec, Quebec, QC Canada; 7 Division of Medical Toxicology, Ronald O. Perelman Department of Emergency Medicine, NYU Grossman School of Medicine, New York, NY USA; 8 Department of Medical and Toxicological Critical Care, Lariboisière Hospital, INSERM UMRS-1144, Paris Cité University, Paris, France; 9 Social Determinants of Health Research Center, Shahid Beheshti University of Medical Sciences, Tehran, Iran; 10 Department of Clinical Toxicology, School of Medicine, Shahid Beheshti University of Medical Sciences, Tehran, Iran; 11 Ottawa, Ontario Canada; 12 Department of Internal Medicine-Division of Nephrology, Lebanese American University - School of Medicine, Byblos, Lebanon; 13 Department of Emergency Medicine, U Mass Memorial Health, U Mass Chan Medical School, Worcester, MA USA; 14 Clinical Toxicology, Guy’s and St Thomas’ NHS Foundation Trust and King’s Health Partners, and Clinical Toxicology, Faculty of Life Sciences and Medicine, King’s College London, London, UK; 15 New South Wales Poisons Information Centre, Sydney Children’s Hospitals Network, Westmead, NSW Australia; 16 Drug Health Services, Royal Prince Alfred Hospital, Sydney, NSW Australia

**Keywords:** EXTRIP, Hemodialysis, CKRT, Poisoning, Ethylene glycol

## Abstract

Ethylene glycol (EG) is metabolized into glycolate and oxalate and may cause metabolic acidemia, neurotoxicity, acute kidney injury (AKI), and death. Historically, treatment of EG toxicity included supportive care, correction of acid–base disturbances and antidotes (ethanol or fomepizole), and extracorporeal treatments (ECTRs), such as hemodialysis. With the wider availability of fomepizole, the indications for ECTRs in EG poisoning are debated. We conducted systematic reviews of the literature following published EXTRIP methods to determine the utility of ECTRs in the management of EG toxicity. The quality of the evidence and the strength of recommendations, either strong (“we recommend”) or weak/conditional (“we suggest”), were graded according to the GRADE approach. A total of 226 articles met inclusion criteria. EG was assessed as dialyzable by intermittent hemodialysis (level of evidence = B) as was glycolate (Level of evidence = C). Clinical data were available for analysis on 446 patients, in whom overall mortality was 18.7%. In the subgroup of patients with a glycolate concentration ≤ 12 mmol/L (or anion gap ≤ 28 mmol/L), mortality was 3.6%; in this subgroup, outcomes in patients receiving ECTR were not better than in those who did not receive ECTR. The EXTRIP workgroup made the following recommendations for the use of ECTR in addition to supportive care over supportive care alone in the management of EG poisoning (very low quality of evidence for all recommendations): i) Suggest ECTR if fomepizole is used and EG concentration > 50 mmol/L OR osmol gap > 50; or ii) Recommend ECTR if ethanol is used and EG concentration > 50 mmol/L OR osmol gap > 50; or iii) Recommend ECTR if glycolate concentration is > 12 mmol/L or anion gap > 27 mmol/L; or iv) Suggest ECTR if glycolate concentration 8–12 mmol/L or anion gap 23–27 mmol/L; or v) Recommend ECTR if there are severe clinical features (coma, seizures, or AKI). In most settings, the workgroup recommends using intermittent hemodialysis over other ECTRs. If intermittent hemodialysis is not available, CKRT is recommended over other types of ECTR. Cessation of ECTR is recommended once the anion gap is < 18 mmol/L or suggested if EG concentration is < 4 mmol/L. The dosage of antidotes (fomepizole or ethanol) needs to be adjusted during ECTR.

**Supplementary Information:**

The online version contains supplementary material available at 10.1186/s13054-022-04227-2.

## Introduction

Ethylene glycol (EG) poisoning is associated with a high likelihood of acute kidney injury (AKI) [1–3] and mortality [4, 5]. In 2020, the US poison control centers reported 6036 calls relating to EG, 586 of which had at least moderate clinical effects and 30 of which resulted in death [6]. Hemodialysis was first reported in the management of an EG-poisoned patient in 1959 [7] and became a critical component of the management of EG poisoning [8]. However, with the advent and wider availability of fomepizole, the indications for extracorporeal treatments (ECTRs), such as intermittent hemodialysis and continuous kidney replacement therapy (CKRT), have evolved and the role of ECTRs is  currently being challenged [9–11].

The EXtracorporeal TReatments In Poisoning (EXTRIP) workgroup is composed of international experts representing diverse specialties and professional societies (Additional file 1: Table S1). Its mission is to provide recommendations on the use of ECTRs in poisoning (http://www.extrip-workgroup.org). The rationale, background, objectives, methodology, and its initial recommendations are previously published [12–14]. The objective of this article is to present EXTRIP’s systematic review of the literature and recommendations for the use of ECTR in patients poisoned with EG. Although diethylene glycol and other alcohols share common characteristics with EG, this review is restricted to EG poisoning.

### Physicochemical characteristics and toxicokinetics

The toxicokinetics of EG are summarized in Table 1. EG, like other alcohols, is a small water-soluble molecule that is quickly and completely absorbed in the gastrointestinal tract. It has negligible protein binding and distributes in total body water. One third of absorbed EG is eliminated unchanged in urine while two-thirds are oxidized in the liver by the enzyme alcohol dehydrogenase (ADH) to glycolaldehyde, which is then rapidly converted to glycolate by aldehyde dehydrogenase [15]. Glycolate is converted to glyoxylate by glycolate oxidase which is the rate-limiting step. Glyoxylate is later metabolized by various pathways to oxalate and non-toxic products such as glycine and α-hydroxy-β-ketoadipate. EG elimination follows first-order pharmacokinetics but a biphasic elimination profile is described [16]. Total body clearance of EG is approximately 100 mL/min, a fourth of which is attributable to kidney clearance and is directly proportional to kidney function [15]. Consequently, the half-life (T_1/2_) of EG is prolonged in patients with a decreased glomerular filtration rate (GFR). Ethanol and fomepizole both compete with EG for ADH, so their administration prevents the metabolism of EG, prolonging the apparent elimination T_1/2_ of EG. Because fomepizole has a stronger affinity and inhibition of ADH compared to ethanol [17, 18], EG elimination T_1/2_ is longer during fomepizole than during ethanol therapy (Table 1).

**Table 1:** Chemical characteristics and toxicokinetics of ethylene glycol

Characteristics		Ethylene glycol	Glycolate	References
Molecular weight (Da)		62	76	
Bioavailability (%)		100 *(rodent data)*	N/A	[402]
Protein binding (%)		Unknown, likely very low	0	[403]
Volume of distribution (L/kg)		0.5–0.8	0.5–0.6	[15, 19, 148, 178, 202, 209, 210, 342, 404–406]
T_1/2_ (Hours)	No antidote, no KI	2–5	2–7	[15, 16, 28, 41, 50, 119, 143, 144, 148, 160, 163, 165, 168, 172, 177, 178, 185, 186, 188, 202, 210, 211, 218, 221, 222, 224, 227, 242, 278, 385, 404–422]
	No antidote, KI	4–8	?	
	Ethanol, no KI	8.5–14	1–3	
	Ethanol, KI	20–40	15–40	
	Fomepizole, no KI	12–18	3–5	
	Fomepizole, KI	40–80	10–15	
Clearance (mL/min)	Non-renal (no antidote)*	60–100	80–85	[15, 143, 172, 178, 210, 404, 406, 413, 414, 423–425]
	Non-renal (with antidote)	5–10		
	Renal (no KI)	20–30	5–60	

*N/A* Not applicable, *KI* Kidney impairment including both *AKI* (acute kidney injury) and *CKD* (chronic kidney disease)

^*^No antidote = neither ethanol nor fomepizole

### Review of ethylene glycol toxicity

EG is the main component of commercial antifreeze and is present in many industrial products including coolants and de-icers. EG itself has minimal toxicity, but its metabolites are responsible for most of the clinical effects; glycolate contributes to the acidemia, while deposition of calcium oxalate crystals in tissues causes AKI and neurological complications [19–21].

The initial clinical manifestations of EG poisoning mimic those of ethanol ingestion, namely inebriation and ataxia. As EG is metabolized, metabolic acidemia appears after a latent period of approximately 3–6 h after ingestion. Thereafter, progressive neurotoxicity (coma, cerebral edema, cranial nerve palsies, and seizures), cardiotoxicity (tachycardia with hypertension or hypotension), respiratory distress, and AKI occur. The incidence of AKI varies between 30 and 70% [22–34]. Multiorgan failure and death can occur at this stage. Cranial nerve palsies, radiculopathy, and other neuropathies may appear several days after ingestion, despite treatment [35–37]. Rarely, brainstem and basal ganglia injuries are reported [38, 39].

A threshold dose for toxicity is poorly defined in humans. Aircraft de-icing workers systemically exposed to an estimated 27 mg/kg from aerosolized EG (≈ 2 mL of pure EG) did not demonstrate any adverse effects [40]. Self-experiments with EG revealed no harm with pure EG ingestions of 10–30 mL [41–43]. In one cohort of 86 unintentional ingestions of < 100 mL EG, all patients survived [44] and only one patient developed mild AKI, although most were treated with ethanol and/or hemodialysis within 3 h of ingestion. The often-quoted lethal dose in an untreated 70 kg adult is 100 mL [45–48], although there are several cases of toxicity and even death below this dose (Additional file 1: Table S7) [37, 49–56]. EG dose is prognostic of outcomes only if there is a delay to treatment [46, 57, 58]. Similarly, a concentration threshold for toxicity is unknown; some sources quote a peak EG concentration > 3.2 mmol/L (20 mg/dL) as a risk for toxicity [59], over which treatment should be initiated [11]. Some authors propose a treatment threshold of 10 mmol/L (62 mg/dL) in asymptomatic patients if base deficit is below 10 mmol/L, based on molar-molar conversion of EG to glycolate in the absence of supporting clinical data [60]; this is not a routine recommendation by most poison centers. Toxicity did not occur in 7 untreated patients who had EG concentrations < 4.8 mmol/L (30 mg/dL) [44, 61, 62]. Reports of toxicity when the EG concentration is < 3.2 mmol/L (20 mg/dL) are exclusively in patients who have already metabolized EG when testing is performed and does not represent peak EG concentrations. Because EG itself causes little toxicity, the EG concentration is poorly predictive of mortality [23, 24, 27, 28, 32, 63–69].

Other than dose and concentration, EG toxicity is modulated by co-ingestion with ethanol because this decreases EG metabolism [25, 29, 69, 70]. Patients in whom medical care is delayed, for example late presentations, develop more toxicity due to a higher concentration of toxic metabolites [23, 63]. Data suggest that a delay of 6–12 h between EG ingestion and treatment initiation is associated with an increased risk of immediate and long-term complications [32, 55, 58], although this was not confirmed in other studies [52, 63, 65].

Complications from EG poisoning are predicted by the concentration of plasma glycolate and associated acid–base disorders [23, 24, 29, 63, 66, 68, 71–73]. The development of AKI is closely correlated with other outcomes including death [29, 31, 32, 52], as AKI is a marker of metabolite-mediated organ injury, and it delays kidney excretion of EG. Death very seldom occurs if AKI is not present [23, 27, 29]. Other clinical markers of mortality are coma [23, 27, 31, 32, 63, 65, 67, 74], respiratory failure [23, 27, 31, 32], hypotension [23, 31], and seizures [31, 63, 65].

With better supportive care, increasing awareness of treatment priorities, widespread use of antidotes, and greater availability of ECTR, mortality from intentional EG poisoning has steadily decreased: The mortality exceeded 80% prior to 1960 [7, 75, 76] and decreased to 30–40% in the 1970s and 1980s [22, 23, 53, 56, 77–79]. This trend has continued to improve during the 1990s [24, 27, 31, 52, 80] with mortality declining to < 10% today [32, 33, 67, 81]. High mortality rates are still reported when antidotes and/or ECTR are not readily available [4, 5, 51, 63, 82, 83].

Persisting sequelae are unusual in survivors. AKI lasts approximately 7–10 days [27, 65, 84, 85] and kidney function returns to baseline in most patients [27, 86] However, there are cases of residual chronic kidney disease (CKD) [80, 87–89] including patients who remain dialysis-dependent [26, 36, 84, 90–92] one year later. Similarly, neuropathy regresses over time although there are cases of chronicity [93–99]. The incidence of these sequelae is unclear but appears to be less than 1% [68].

Standard care for patients with an EG exposure includes assessment for and treatment of abnormalities of the airway, breathing and circulation, correction of acid–base disorders, and ADH-inhibitor therapy (Additional file 1: Table S2). Ethanol and fomepizole decrease EG metabolism through competitive inhibition of ADH, and both prevent toxicity and death in EG-poisoned animals [18, 100–103]. Ethanol has been used as an antidote in humans since the 1960s [104]. Fomepizole was approved in the USA in the late 1990s [28] and has largely replaced ethanol as the antidote of choice for EG poisoning in many countries [81]. Thiamine and pyridoxine are used to facilitate the conversion of glyoxylate to non-toxic metabolites rather than oxalate, but their clinical utility has never been determined.

## Methods

The workgroup developed recommendations on the use of ECTR in EG exposures, following the EXTRIP methodology previously published [13], with modifications, updates, and clarifications. The methods and glossary are presented in full in the Supplementary Material, including the PRISMA checklist (Additional file 1: Table S3), dialyzability criteria (Additional file 1: Table S4), quality assessment of individual toxicokinetic studies (Additional file 1: Table S5), and evidence (Additional file 1: Table S6). These data were assessed according to GRADE methodology (Additional file 1: Figure S1) which also informed the voting process for recommendations (Additional file 1: Figure S2). Cases in which ECTR was performed solely for anuria or related complications were excluded.

Complementary searches were also performed to answer specific questions regarding 1) dialyzability of EG metabolites, 2) dialyzability of ethanol/fomepizole, 3) dialyzability of pyridoxine and thiamine.

## Results

**Fig. 1:**
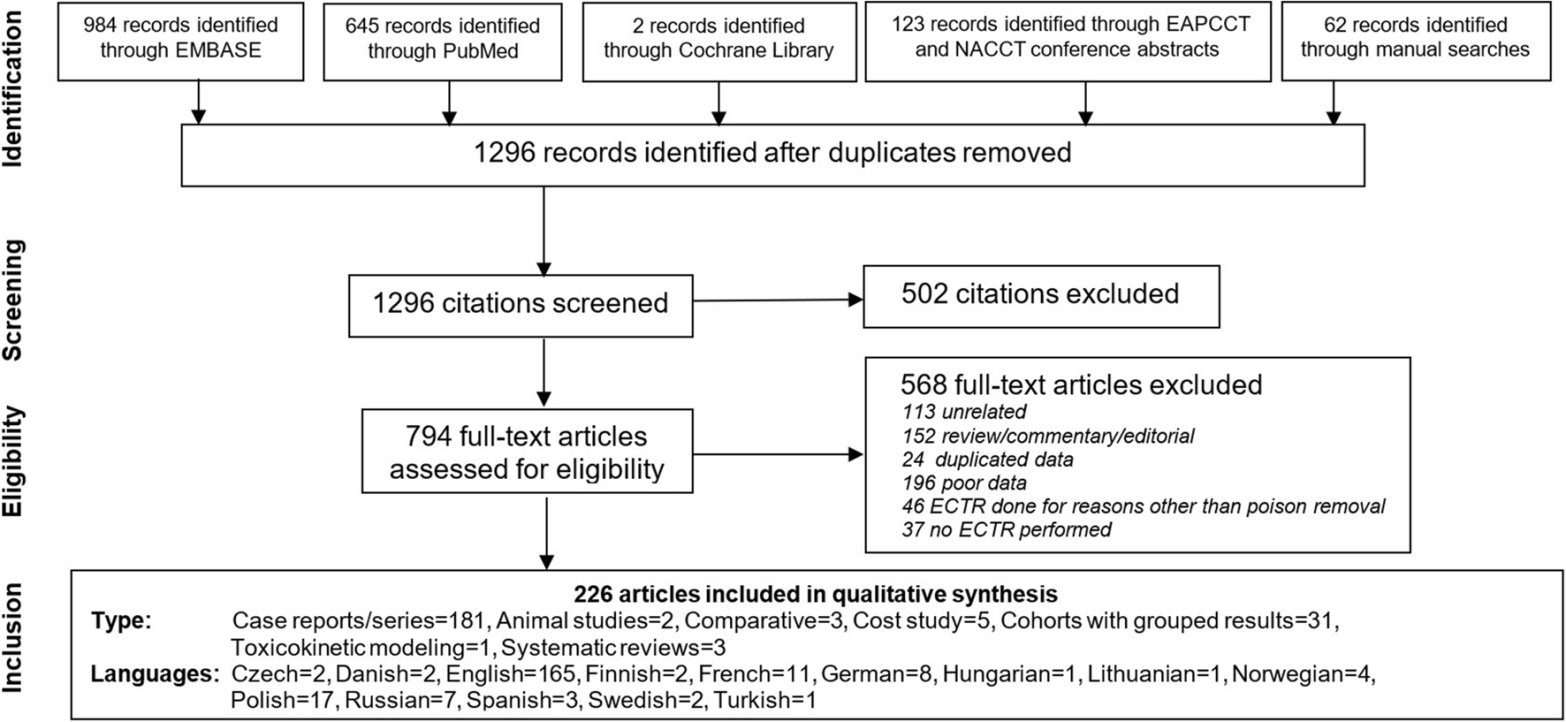
Article selection

Results of the primary literature search (first performed on March 1, 2019, and last updated February 1, 2022) are presented in Fig. 1.

A total of 1296 articles were identified after removal of duplicates. In the final analysis, 226 articles were included for qualitative analysis, including two animal experiments [105, 106], five cost-evaluation studies [107–111], 31 cohort studies with pooled data [5, 22–24, 27, 28, 31–34, 56, 58, 67, 69, 73, 84–87, 112–123], one toxicokinetic modeling study [124], 181 case reports and case series with individual patient-level data [7, 25, 26, 29, 30, 37–39, 44, 51, 53, 64, 66, 70, 80, 89, 104, 125–288], three comparative studies [52, 55, 63], and three systematic reviews [289–291]. No randomized controlled trials were identified. One fourth of all selected articles were in languages other than English.

Other articles were obtained, although they were not part of the initial systematic literature search, namely relating to dialyzability of EG metabolites [292–306], dialyzability of ethanol [307–317], dialyzability of fomepizole [318–323], and dialyzability of pyridoxine and thiamine [324–341]. Data from publications reporting the same subjects were merged but the citations were only counted once in the systematic review, e.g., [15, 28, 211] and [19, 147, 151, 342].

### Summary of the evidence: dialyzability

#### Dialyzability of EG

EG and its metabolites have characteristics of dialyzable poisons [343], namely small size, high water solubility, absence of protein binding, and a low volume of distribution (Table 1). EG clearance with high-efficiency intermittent hemodialysis is similar to that of urea [133, 172], approximating plasma flow and can surpass 200 mL/min (Table 2) [15, 148, 209]. EG clearance from hemodialysis increases total clearance by at least 800%, compared to endogenous clearance (assuming adequate ADH blockade). As for other small molecules, increasing blood and effluent flow and using a dialyzer with a higher surface area will increase EG clearance [163]. Mass removal of EG can exceed 100 g during a 6-h hemodialysis [148, 202, 287, 342].

**Table 2:** Toxicokinetics of ethylene glycol and metabolites during various extracorporeal treatments

Poison	ECTR	TK (Poisoning)						References
		T_1/2_ (hours)*			ECTR clearance (mL/min)**			
		Median	*N*	Range	Median	*N*	Range	
Ethylene glycol	PD	13.2	4	6.1–18.6	7.3	3	6.5–17.2	[150, 152]
	CKRT	6.3	3	3.2–10.7	72	1	14.1–130	[196, 197, 252, 287]
	HD	2.9	88	0.6–27.1	163	42	14–260	[15, 23, 38, 67, 115, 123, 133, 140, 145, 148, 149, 156, 159, 160, 163, 166, 168, 171, 172, 178, 183, 185, 188, 189, 192, 198, 202, 209, 210, 214, 216, 219, 224, 227, 244, 270, 273, 278, 288, 342]
	HP				26	1		[143]
Glyoxylate	HD-HP	1.3	1		71	1		[176]
Glycolate	HD	2.4	14	0.4–4.4	146	9	63–205	[164, 178, 185, 211, 224, 426]
Oxalate	HD/HDF***				> 100	26		[300, 301, 303, 304]
	PD				< 8	54		[293, 295–297, 302, 303]

*ECTR* Extracorporeal treatment, *PD* Peritoneal dialysis, *CKRT* Continuous kidney replacement therapy, *HD* Hemodialysis, *HP* Hemoperfusion, *HDF* Hemodiafiltration

^*^Regardless of antidote

^**^The panel noted several cases where clearance was calculated using plasma concentration and blood flow [160, 163, 164, 168, 178, 342] which will overestimate ECTR clearance. When noted, clearances were recalculated with the given or estimated hematocrit

^***^Assuming new technology

Continuous and hybrid techniques (e.g., CKRT, sustained low-efficiency hemodialysis, extended daily dialysis) provide inferior EG clearances and mass removal compared to standard intermittent hemodialysis, due to their lower blood and/or effluent rates (Table 2) [196, 287]. Nevertheless, these techniques still achieve a substantial increase in total EG clearance. As EG has negligible protein binding, no advantage would be expected from therapeutic plasma exchange, liver support devices, and hemoperfusion; in one case, EG clearance during hemoperfusion only reached 50 mL/min and quickly decreased because of extensive cartridge saturation [143]. Low-efficiency techniques like peritoneal dialysis have modest effect on removal of both EG or metabolites [128, 132, 139, 303], but will provide a clearance that exceeds endogenous clearance in the presence of AKI and adequate ADH blockade. No toxicokinetic data exist for exchange transfusion. After completion of ECTR, a rebound increase in EG concentration was observed in 21% of the cohort and the median magnitude of this rebound was 30% of the immediate post-ECTR concentration; in one case [144], the rebound was substantial (200%).

As mentioned, once ADH is blocked by ethanol or fomepizole, endogenous EG clearance is at best 30 mL/min, which is modest relative to extracorporeal EG clearances with modern-day efficient ECTRs (Table 2) [148, 192, 209, 211]. For this reason, dialyzability was not graded based on kidney function. Although most of the toxicokinetic data are dated with technology considered substandard today, EG was considered “dialyzable” with intermittent hemodialysis (level of evidence = B, Table 3), “moderately dialyzable” with CKRT (level of evidence = D), “slightly dialyzable” with peritoneal dialysis (level of evidence = C), and “slightly dialyzable” with hemoperfusion (level of evidence = D). No data exist on intermittent hemodiafiltration, but it is expected to perform as well as hemodialysis, based on achievable urea clearance. In the rare scenario in which no antidote is available, high-efficiency hemodialysis would increase endogenous clearance at least 100% [278], i.e., “moderate” dialyzability.

**Table 3:** Final toxicokinetic grading of ethylene glycol and glycolate during extracorporeal treatments

Poison	Toxicokinetic grading	Number of patients fulfilling toxicokinetic grading			
		Hemodialysis	Peritoneal dialysis	Continuous kidney replacement therapy	Hemoperfusion
Ethylene glycol	Dialyzable	19		1	
	Moderately dialyzable	6			
	Slightly dialyzable	1	2	1	1
	Not dialyzable		1		
	*Final grading and level of evidence*	*Dialyzable (B)*	*Slightly dialyzable, (C)*	*Moderately dialyzable, (D)*	*Slightly dialyzable, (D)*
Glycolate	Dialyzable	3			
	Moderately dialyzable	1			
	Slightly dialyzable				
	Not dialyzable				
	*Final grading and level of evidence*	*Dialyzable (C)*			

#### Dialyzability of ethylene glycol metabolites

EG metabolites have physicochemical characteristics indicative of being dialyzable, as is confirmed by data; however, caution is required when grading dialyzability according to half-life comparison, during and off ECTR, as these may be influenced by ongoing variable production of metabolites, especially when ADH inhibition is inadequate.

*Glycolate*: High glycolate clearance (> 150 mL/min) and high mass removal of glycolate (up to 50 g) are reported during hemodialysis [19, 164, 211]. There are reports of glycolate concentrations increasing modestly during ECTR suggesting that production surpassed elimination and that ADH blockade was inadequate [165]. Glycolate can also reaccumulate after ECTR, especially if ADH blockade is not continued [181, 185, 344]. Based on 4 patients in whom dialyzability could be assessed, glycolate was rated as “dialyzable” with hemodialysis (level of evidence = C) (Table 3).

*Oxalate*: Oxalate was detected in blood in only 4 of the 10 patients in whom it was measured, in concentrations at least 20 times lower than those observed for glycolate [150, 241, 250, 252]. In one report, three sessions of hemodialysis removed on average of 380 mg of oxalate, although dialyzability could not be estimated [131]. From studies in dialysis-dependent CKD patients with primary or secondary oxalosis, oxalate clearance surpasses 150 mL/min with hemodialysis and hemodiafiltration [301, 303–305], which is at least 300% more than the kidney elimination capacity [292, 297]. Oxalate clearance in peritoneal dialysis is consistently less than 8 mL/min [139, 293, 295, 296, 303].

*Glyoxylate*: Glyoxylate extracorporeal clearance by hemoperfusion–hemodialysis was 71 mL/min in one patient [176].

#### Dialyzability of ethanol/fomepizole

Both ethanol [316] and fomepizole [318, 322, 345] are extensively removed by ECTR. If the dose of either of these antidotes is not increased during the ECTR session, a risk of inadequate inhibition of EG metabolism during ECTR exists. The elimination T_1/2_ of ethanol and fomepizole during hemodialysis and hemodiafiltration ranges between 1.5 and 3.0 h [308, 310, 312, 313, 315, 319, 321, 345, 346], and ECTR clearance surpasses 100 mL/min [198, 202, 272, 308, 310, 319, 345]. Considerably lower amounts of these antidotes are removed during CKRT [272, 320, 347, 348], and particularly during peritoneal dialysis [307, 311].

#### Dialyzability of pyridoxine and thiamine

*Pyridoxine*: In vivo clearance of pyridoxal-5’-phosphate with cellulose filter membranes averaged 170 mL/min in 6 subjects [328], while it was < 1 mL/min with peritoneal dialysis [329].

*Thiamine*: Thiamine concentration decreased between 5 and 40% during hemodialysis, but extracorporeal clearance was not calculated [325, 332, 336, 340].

### Summary of the evidence: pre-clinical data

Three animal studies with group comparisons were identified [105, 106, 349]. In one experiment, an LD_400_ dose of EG was given to 23 dogs; 13 were treated with intravenous NaHCO_3_ and 10 were treated with a single session of hemodialysis for 20–24 h. All died in the NaHCO_3_ group while two died in the hemodialysis group (*p* < 0.0001), suggesting a beneficial effect of hemodialysis [105]. In one experiment of six EG-poisoned dogs, five received 3 h hemoperfusion within 5 h of poisoning and one was a control. All died and no improvement was seen from hemoperfusion [106].

### Summary of the evidence: clinical data

#### Comparative data

We identified one retrospective study in which supportive care with ECTR (*n* = 28) was directly compared to supportive care alone (*n* = 28, Table 4) [63]. The mortality was the same in both groups (8/28 = 28.6%), although the ECTR group was sicker at baseline (higher EG dose, lower pH or HCO_3_^−^ concentration, higher anion gap, greater percent with coma and respiratory failure, longer delay to admission, longer delay to antidotal therapy). An important limitation is that only about half of the entire cohort received antidotal therapy (ethanol).

**Table 4:** Included studies comparing the effect of extracorporeal treatments vs no extracorporeal treatments

References	Location, Year of recruitment	Study design (analysis)	Number and type of patients	Intervention	Comparator	Reported outcomes and conclusion	Baseline comparison	Noticeable limitations, and overall interpretation
Grigorasi [63] (published as a conference abstract in 2018)	Romania; January 2012–October 2017	Retrospective multicenter chart review in the context of a large EG poisoning epidemic; (cooperative study between regional hospitals, a university hospital, and a poison information center)	(*n* = 56) confirmed by blood EG measurement. All had plasma creatinine > 88 µmol/L (1 mg/dL) on admission Global severity of included population: death occurred in 16/56 (29%) of patients	HD (*n* = 28) 18/28 patients (64%) also received the antidote ethanol	No HD (*n* = 28) 14/28 patients (50%) also received the antidote ethanol	Comparable mortality (8/28 died in each group) and CKD between groups Neither delay to admission, dialysis use, HCO_3_^−^ concentration or ingested alcohol volume were related to death Death was more likely in patients with seizures, coma, severe acidosis (pH 7.05) or AKI on admission;	Patients who received HD were more symptomatic on admission, had higher blood EG concentration, lower initial pH and HCO_3_^−^, higher peak creatinine at 24 h, and had a longer ingestion-to-admission time as well as door-to-antidote time, compared to group who did not receive HD group Co-ingestion with ethanol was comparable between the 2 groups	Comparable mortality between the groups despite a clear confounding-by-indication bias (HD group sicker on admission than the no-HD group) supports a potential beneficial effect of HD, but the low usage of ethanol in the cohort and a small sample size lessen the generalizability of these findings
Lung [32]	California Poison Control System; CA, USA; January 1999–December 2008	Retrospective chart review (non-consecutive)	(*n* = 121) confirmed by blood EG measurement Global severity of included population: Death (*n* = 9, 7.4%) or AKI requiring dialysis for more than 3 days (*n* = 50, 41.3%)	HD (*n* = 102)	No HD (*n* = 19)	Mortality and prolonged AKI were associated with the use of HD, 58/59 (98.3%) vs 43/62 (69.4%) in those who survived without AKI	Patients who received HD had worse indices of illness: lower initial pH, higher initial and peak plasma creatinine	Confounding-by-indication limits conclusions from the data Outcomes were not adjusted for pH despite it being a strong predictor of worse outcome
				97.5% of the cohort (118/121) received an antidote: 65 received fomepizole, 29 received ethanol and 24 received both. Antidotes were administered within 3 h in half of the patients				
Swiderska [5]	Poland Health Services; Poland; year 2010	Retrospective chart review	(*n* = 174) identified through ICD-10 coding reported by medical facilities to the National Health Fund Global severity of included population: deaths = 47/174 (23.6%)	ECTR (*n* = 98)	no ECTR (*n* = 78)	Comparable mortality between groups: mortality in patients not treated with ECTR = 21/78 (26.9%) vs 26/98 (26.5%) in those treated with ECTR	No comparison at baseline	The groups were not compared at baseline, limiting conclusions from the data
				Antidote was recommended but not known if it was given				
White [118]	US poison centers USA; 1995–2005	Retrospective chart review of prospectively collected information	(*n* = 3623) with intentional, suspected suicidal ingestion of EG Global severity of included population: Deaths (*n* = 1 47/2773, 5.3%) and “major effect” (1089/2773, 39.3%)	HD (*n* = 1611)	no HD (*n* = 2012)	Receiving HD increased the odds of severe outcomes (death or major effect) and being admitted to a critical care unit, while receiving an antidote was associated with a decrease in the odds of severe outcomes	Logistic regression model to identify variables associated with severe outcomes (life-threatening major effects or fatal cases): adjusted for age, time (beginning vs end of study), gender, antidote administered, HD, critical care and bittering agent. Logistic model included *n* = 1790 (numbers HD vs no HD not reported). HD: adjusted OR 16.93 (13.16 to 21.77). Also, statistically significant: male gender, antidote administered, critical care	Confounding-by-indication (all variables associated with severe outcomes are also associated with severity of illness) limits conclusions from the data
				Antidote (fomepizole, ethanol or both) was administrated in a total of 2205 patients (60.9%)				
Krenova [44]	Czech Toxicological Information Center, Czech Republic; 2000–2004	Retrospective chart review	(*n* = 86) hospitalized for an accidental EG ingestion (1–3 swallows), for whom the amount of EG ingested was known and/or the blood EG concentration were measured Global severity of included population: no deaths, AKI (10%), CNS depression (9%)	HD (*n* = 17) 15/17 patients (88%) received ethanol: 8 as first aid and 15 as antidote in hospital	No HD (*n* = 69) 63/69 patients (91%) received ethanol: 15 as first aid and 59 as antidote in hospital	Comparable mortality and CKD between groups (no mortality; one case of temporary deterioration of baseline CKD in the HD group)	No comparison at baseline (only descriptive reporting)	Confounding-by-indication and very few events (CKD or mortality) limits conclusions from the data
Porter [29]	Kentucky, USA; 1997–2000	Retrospective chart review	(*n* = 41) admissions (in 39 patients), confirmed by initial plasma EG and glycolate concentrations Global severity of included population: Deaths (*n* = 8/41, 19.5%)	Ethanol + HD (*n* = 3 3)	Ethanol + no HD (*n* = 8)	Overall, receiving HD was associated with a reduction in the risk of death at the univariate analysis (mortality of 5/33 (15.2%) in HD group vs 3/8 (37.5%) in the no-HD group) (RR: 0.30 95%CI (0.10–0.87) The authors concluded that HD might not be required in patients with glycolate less than 8 mmol/L (correlated surrogates = anion gap less than 20 mmol/L or pH more than 7.30) with adequate antidote therapy since they are not likely to develop AKI	A stratified analysis according to severity in the survivor group is presented (based on presence of AKI and plasma glycolate), but no matched or adjusted analysis on baseline data	Few patients treated without HD and there was marked variability in clinical severity (too sick or barely sick) in the HD group The authors’ conclusions regarding the predictive value of glycolate is confounded since 10/17 patients with glycolate less than 8 mmol/L received HD Critical lack of detail
Donovan [107] (Conference abstract)	Philadelphia, USA. Reported in 1998, timing of recruitment unclear	Retrospective single-center chart review	(*n* = 4) Global severity of included population: no deaths (0%)	HD + fomepizole (*n* = 2)	no ECTR, fomepizole alone (*n* = 2)	All patients had good outcomes without progression of acidosis or development of AKI The LOS was longer in patients who only received fomepizole. There was no direct correlation between costings and use of HD vs fomepizole alone	No comparison at baseline (only descriptive reporting)	Too underpowered to make any meaningful comparison (either of baseline characteristics or clinical outcomes). Cost analysis is dated and not likely valid outside this institution
Stompor [25]	Krakow, Poland; 1990–1994	Retrospective single-center (department of toxicology) chart review	(*n* = 36) Global severity of included population: Death (*n* = 18/36; 50%)	ECTR (*n* = 24) (one HD session, 3 to 9 h after admission, for 6–16 h) Unknown use of antidotes.	no ECTR (*n* = 12). Unknown use of antidotes.	Overall, receiving ECTR was associated with a non-statistically significant increase in mortality (14/24 (58.3%) in the ECTR group vs 4/11 (36.4%) in the no ECTR group), but was associated with the presence of AKI (16/24 (66.7%) vs 1/11 (9.1%))	When comparing the no ECTR group to the ECTR group, no statistical difference was observed in ethanol co-ingestion, pH, HCO_3_^−^, base excess, or 24 h urine output. However, plasma EG concentration was higher in the ECTR group compared to the no ECTR group (153.5 mg/dL vs 15.2 mg/dL, respectively)	Difficult to interpret: no difference in clinical outcomes between the two groups despite the ECTR group having higher plasma EG concentrations and AKI, but likely underpowered. No mention of antidotal therapy
Karlson-Stiber and Hylander [24, 65]	Sweden; 1987 Two publications with overlapping data	Retrospective chart review (telephone consultation to a poison center and chart review of county and university hospitals)	(*n* = 36) (charts were reviewed in only 17 patients) Diagnosed by plasma EG concentration in 24 patients, and by urinary calcium oxalate crystals in 6 patients Global severity of included population: Death (*n* = 6/36, 16.7%)	ECTR (*n* = 29, 28 HD, 1 PD alone)	no ECTR (*n* = 7)	Comparable mortality between groups: mortality of 4/29 (13.8%) in the ECTR group vs 2/7 (28.7%) in the no ECTR group	No comparison at baseline reported	Both groups were not compared at baseline and the study is underpowered which limits conclusions from the data Furthermore, confounding-by-indication might have biased the study since the decision to not perform ECTR was due to mild symptoms or to a poor clinical status
				88% of patients received IV ethanol				

*ECTR* Extracorporeal treatment, *PD* Peritoneal dialysis, *HD* Hemodialysis, *HDF* Hemodiafiltration, *LOS* Length of stay, *AKI* Acute kidney injury, *IV* Intravenous, *EG* Ethylene glycol, HCO_3_^−^ Bicarbonate

Cohorts in which the effect of ECTR could be analyzed were extracted and analyzed (Table 4) [5, 24, 25, 29, 32, 44, 107, 118]. Unfortunately, none of these studies were designed to appropriately determine the effect of ECTR, and all contained critical methodological flaws that preclude meaningful conclusions. These limitations include small sample size, retrospective design (except for poison control data which have other limitations), unclear patient selection, variable definitions of EG poisoning (reported amount ingested vs laboratory testing), unreported baseline characteristics and exposure details (especially co-ingestion with ethanol, volume EG ingested, time from EG ingestion to admission, antidote used), imprecise indications for ECTR, and confounding-by-indication, i.e., ECTR is preferentially used in those with more severe clinical features. Some cohorts included patients that could have been plausibly managed without ECTR [44]. One study suggested that hemodialysis increased the odds of severe outcomes 17-fold [118], after adjusting for age, gender, year, addition of bittering agent, administration of antidote and admission to critical care. This likely quantifies the extent of confounding-by-indication and selection bias in real-life presentations. There were studies that compared types of ECTR [5, 24, 52, 55] (Additional file 1: Tables S8 and S9), number of treatments [65], or the impact of time to ECTR initiation on clinical outcomes [32, 56] (Additional file 1: Tables S8 and S9). No meaningful conclusions can be inferred from these studies, as the same major limitations apply. Therefore, clinical data were not considered suitable for inclusion in a meta-analysis comparing ECTR to no ECTR.

#### Clinical cases

A total of 446 cases had sufficient patient-level data, the summary of which are shown in Table 5. Most patients received ethanol rather than fomepizole for ADH blockade, reflecting older literature or country of origin. Intermittent hemodialysis was by far the most used ECTR. Fifty-four percent of patients received more than one ECTR session (usually for management of uremia), comparable to other cohorts [32]. Acidemia was corrected quickly in most cases receiving high-efficiency hemodialysis, usually within four hours. Peritoneal dialysis was switched to hemodialysis because of clinical failure in one patient [160]. Mortality from the entire cohort was 18.7% and the median time to death was 96 h after ingestion. Some patients with massive ingestions (> 1 L) or very high EG concentrations (> 200 mmol/L or > 1240 mg/dL) survived [123, 214, 238, 274, 281, 282], as did some with extreme acid–base abnormalities (e.g., pH < 6.60 or HCO_3_^−^  < 2 mmol/L) [25, 26, 29, 104, 180, 190, 191, 215, 223, 226, 236, 244, 248, 254, 257, 264, 276]. As suggested in one review [71], poor outcomes were infrequent when the glycolate concentration is < 12 mmol/L or the anion gap (with potassium, calculated as Na^+^ + K^+^– Cl^- ^– HCO_3_^−^) is < 28 mmol/L (Additional file 1: Table S10); three such patients who received ECTR died, in two cases there were limited details reported [51], and one died without receiving an ADH inhibitor [126]. Mortality in patients who had an anion gap over 28 mmol/L was much higher (20.4%).

**Table 5:** Clinical summary of included cases with ethylene glycol poisoning

		All cases (*n* = 446)	*Early* EG poisoning (glycolate concentration ≤ 12 mmol/L or an anion gap ≤ 28 mmol/L; *n* = 84)^**&**^	*Late* EG poisoning (glycolate concentration > 12 mmol/L or an anion gap > 28 mmol/L; *n* = 147)^**&**^
Patient characteristics	Age (Years)	42 [28, 52]	42 [28, 54]	45 [28, 55]
	Male gender	80%	67%	84%
Poisoning information	Ingested dose (mL)	250 [150, 500]	300 [120, 600]	300 [200, 946]
	Ethanol co-ingestion	55%	61%	36%
	Time from ingestion to admission (hours)	10 [4, 18]	6 [2, 11]	12 [7, 22]
	Peak ethylene glycol concentration (mmol/L)	17.6 [6.9, 41.9]	26.8 [10.5, 57.0]	29.4 [9.0, 56.4]
Signs/Symptoms	Altered mental status*	*n* = 237	*n* = 42	*n* = 115
	Coma*	*n* = 127	*n* = 14	*n* = 62
	Cerebral edema*	*n* = 10	*n* = 0	*n* = 8
	Seizure*	*n* = 44	*n* = 4	*n* = 26
	Hypotension*	*n* = 26	*n* = 0	*n* = 11
	Acute kidney injury (KDIGO stage 2 or 3 AKI)	*n* = 295	*n* = 32	*n* = 132
	Lowest pH	7.08 [6.89, 7.23]	7.30 [7.16, 7.38]	7.00 [6.89, 7.11]
	Lowest HCO_3_^−^, (mmol/L)	6.9 [3.2, 13.3]	16 [11, 21]	5 [3, 7]
	Calcium oxalate crystals in the urine*	*n* = 85	*n* = 14	*n* = 14
	Lowest total calcium concentration (mmol/L)	2.1 [1.9, 2.5]	2.1 [1.9, 2.3]	2.0 [1.9, 2.5]
	Anion gap (mmol/L; with potassium)**	32 [25, 39]	22 [16, 26]	37 [32, 43]
	Osmol gap	40 [27, 82]	48 [27, 97]	38 [26, 78]
	Nadir base excess (mmol/L)	− 25 [− 19, − 32]	− 17 [− 11, − 18]	− 24 [− 21, − 31]
	Peak glycolate concentration (mmol/L)	15.9 [5.1, 22.6]	1.6 [0, 7.4]	21.2 [16.4, 25.0]
Other treatments	Gastric lavage*	*n* = 47	*n* = 13	*n* = 11
	Ethanol (any type)*	*n* = 246	*n* = 49	*n* = 81
	Fomepizole*	*n* = 69	*n* = 18	*n* = 35
	Thiamine*	*n* = 56	*n* = 8	*n* = 26
	Pyridoxine*	*n* = 54	*n* = 8	*n* = 25
	Sodium bicarbonate*	*n* = 196	*n* = 17	*n* = 70
	Vasopressors*	*n* = 25	*n* = 1	*n* = 12
	Mechanical ventilation*	*n* = 155	*n* = 24	*n* = 77
ECTR	Hemodialysis	86.5%	91.7%	91.6%
	Hemoperfusion	0.2%	0%	0%
	Exchange transfusion	0.2%	0%	0%
	Continuous kidney replacement therapy	4.3%	4.8%	6.1%
	Slow low-efficiency daily dialysis	0.2%	0%	0.7%
	Peritoneal dialysis	2.7%	0%	0.7%
	More than 1 ECTR	5.9%	3.6%	1.4%
	Time from admission to ECTR (hours)	6 [3, 12]	6 [3, 10]	6 [3, 10]
	Time from ingestion to ECTR (hours)	20 [12, 30]	13 [6, 24]	18 [13, 24]
Outcome	Hospital stay (days)	16 [7, 23.0]	8 [2, 15]	18 [9, 26]
	Intensive care unit stay (days)	5 [3, 11]	5 [2, 8]	3 [3, 8]
	Chronic kidney disease sequelae	16.8%	7.4%	18.8%
	Dialysis-dependent chronic kidney disease sequelae	2.9%	1.2%	5.1%
	Central nervous system sequelae	3.3%	0%	4.3%
	Time requiring kidney replacement therapy for acute kidney injury (days)	9 [3, 14]	7 [2, 16]	12 [8, 22]
	Time of serum creatinine concentration normalization after AKI (days)	21 [7, 40]	5 [1, 24]	21 [11, 40]
	Death	18.7%	3.6%	20.4%
	Time to death (hours)	96 [24, 264]	No data	72 [31, 276]

Data were expressed as medians and interquartile range when applicable,

*AKI* acute kidney injury, *ECTR* Extracorporeal treatments, *HCO*_*3*_^−^ bicarbonate concentration, and *KDIGO* Kidney Disease Improving Global Outcomes

^*^when stated to be present

^**^The anion gap was calculated Na^+^ + K^+^- HCO_3_^−^- Cl^−^. If calculated without K^+^, 4 mmol/L was added. If no mention, 2 mmol/L was added

^&^There were 215 other patients in whom neither the anion gap nor glycolate concentrations were reported, so they are not included here

Several complications occurred during ECTR although in most cases, these can be attributed to EG poisoning rather than the procedure itself. Complications assessed as likely related to ECTR included hypotension [7, 127, 130, 153, 166, 181, 258, 265, 275, 276], bleeding related to heparin [141], catheter-related thrombosis [291], catheter-related bacteremia [166, 183], cardiac arrest [167], and death [153, 155]. ECTR can potentially increase intracranial pressure [350–352] and may have aggravated EG-related cerebral edema leading to seizures [141, 177, 178]. Some authors mentioned concerns related to rapid fluid and solute shifts during ECTR, although it is unclear if these resulted in injury [178, 219]. In one cohort of 72 patients receiving hemodialysis for toxic alcohol poisoning (34 for EG), 20 patients experienced a hemodialysis-related adverse reaction including three cases of hypotension and one case of arterial tear during catheter insertion leading to internal bleeding, shock, and cardiac arrest (the patient eventually recovered) [353].

Persistent sequelae were seldomly reported in case reports and observational cohorts. The median duration of kidney replacement therapy for patients who developed AKI was 9 days [IQR 3,14], while the median duration of serum creatinine concentration elevation was 21 days [IQR 7,40], similar to results in published cohorts [23, 25, 27, 65, 84, 85]. Some degree of CKD was present in 16.8% of patients and 2.9% remained dialysis-dependent; however, in most cases, these were noted on hospital discharge and the duration of follow-up, when reported, was often short, so it is expected that these numbers are overestimated. On extended follow-up, < 1% of patients remained dialysis-dependent at 6 weeks and < 5% had CKD at 6 months [30, 52, 65, 68, 86], although there are rare reports of patients who remained dialysis-dependent after 1 year [26, 36, 84, 90–92]. Among other sequelae, there were rare cases of anoxic brain injury, basal ganglia injury, and irreversible cranial nerve palsies [37–39, 80, 94, 128, 151, 201, 215, 221, 254, 354].

#### Cost analysis of ECTR

In patients presenting prior to the development of acidemia or AKI, ECTR may shorten length of stay and associated morbidity due to nosocomial complications and reduce overall healthcare costs. Studies (mostly performed in the USA) report a cost advantage when hemodialysis is added to fomepizole, especially at higher EG concentrations (Table 6) [107–111, 227, 267, 355]. However, these data are dependent on many factors, including health care delivery model, patient location (medical ward vs high-dependency unit), type of ECTR, initial concentration of EG, need for transfer to another institution, and cost of fomepizole, all of which vary between countries and institutions, and are therefore not generalizable.

**Table 6:** Summary of studies evaluating costs of ECTR vs no ECTR in ethylene glycol poisoned patients

References	Population	Conclusion
Ellsworth, 2011 [109]	Average expected patient charges in 1 institution, USA	For [EG] > 8.1 mmol/L HD (1 session) + Fomepizole (2 doses): $**4,823 and 24 h ICU LOS** Fomepizole (3 doses): $**5,631 and 8 h LOS**
Donovan, 1998 [107]	Actual hospital charges of 4 adult patients in 1 institution, within 1 month, USA	2 patients receiving HD + Fomepizole: **$15,616 and $24,315 for LOS of 2.5 days** 2 patients receiving Fomepizole alone: **$30,072 and $16,790 for LOS of 4.5 and 3.5 days**
Cannarozzi, 2010 [108]	Expected hospital charges, USA	Weight < 75 kg: **Costs of Fomepizole + HD > Fomepizole at all [EG], except [EG] = 81–97 mmol/L** Weight 75–100 kg and [EG] = 0–48 mmol/L: **Costs of Fomepizole + HD = Fomepizole** Weight 75–100 kg and [EG] > 48 mmol/L: **Costs of Fomepizole + HD < Fomepizole** Weight > 100 kg and [EG] > 12.1 mmol/L: **Costs of Fomepizole + HD < Fomepizole**
Darracq, 2013 [267]	Expected hospital costs in same adult patient, USA	Fomepizole + HD = **$2,576 for 24 h LOS** Fomepizole = **$4,246 for estimation 48 h LOS**
Wiles, 2014 [110]	Direct costs based on US national statistics, USA	For [EG] < 29 mmol/L: Fomepizole + HD = **$15,054** Fomepizole = **$15,657**
Vasavada, 2003 [227]	Cost estimates based on 1 patient, USA	Ethanol + HD + ICU: $**3,368** Fomepizole + HD + intermediate ward: **$3,804** Fomepizole alone + intermediate ward: **$5,897**
Boyer, 2001 [355]	Expected hospital costs in 1 patient, USA	Fomepizole + ward admission: **$9,524** Ethanol + ICU + Mechanical ventilation + HD: **$15,723**
Roberts, 2019 [111]	Expected costs, based on local estimates, Australia	[EG] ≤ 8 mmol/L: **Costs of Fomepizole + ward < ICU + CKRT** [EG] > 8 mmol/L: **Costs of Fomepizole + HD + ward < Fomepizole + ward < Fomepizole + CKRT + ICU**

Bold text was inserted to highlight the result

*HD* Hemodialysis, *ICU* Intensive care unit, *EG* Ethylene glycol, [EG] Blood ethylene glycol concentration, *CKRT* Continuous kidney replacement therapy, *LOS* length of stay

All presented currencies are in US$, as reported in the studies

#### Clinical data informing the evidence table

The evidence table is shown in Table 7. It was not possible to reliably populate the evidence table for patients with advanced or late EG poisoning (e.g., glycolate concentration > 12 mmol/L or anion gap with potassium > 28 mmol/L) because very few controls (i.e., not treated with ECTR) exist. The exceptions are patients i) too sick to undergo hemodialysis; ii) who died before hemodialysis was initiated; iii) in whom EG poisoning was not diagnosed; or iv) when ECTR was unavailable [85, 356, 357]. For obvious reasons, these patients are not adequate controls. Patient cohorts published before hemodialysis became widely available report extremely high mortality, although this could also be explained by inadequate standard care and absent antidotal therapy. A prospective randomized trial of ECTR in late EG poisoning would not be considered ethical because of a lack of clinical equipoise. (It is assumed that ECTR would produce a large survival effect.)

**Table 7:** Evidence profile table: ECTR + standard care compared to standard care in patients with “early” ethylene glycol poisoning^#^

Certainty assessment							Summary of findings				Importance
№ of studies	Study design	Risk of bias	Inconsistency	Indirectness	Imprecision	Other considerations	ECTR + standard care*	Standard care (controls)	Impact	Certainty	
**Inpatient mortality**											
Early EG poisoning *n* = 2 ^a^	Observational studies	Very serious^b^	Not serious	Serious^c^	Serious^d^	Publication bias strongly suspected^e^	This systematic review: 3.6%	Fomepizole: 0.0% [358]	No reduction with ECTR	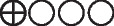 VERY LOW	CRITICAL
								Ethanol: 0.0% [358]	No reduction with ECTR		
								No antidote: Expected high	Very probable reduction with ECTR		
**Dialysis dependence at 3 months**											
Early EG poisoning *n* = 2 ^a^	Observational studies	Very serious ^b^	Not serious	Serious ^c^	Serious ^d^	Publication bias strongly suspected ^e^	This systematic review: 1.2%	Fomepizole: 0% [358]	No reduction with ECTR	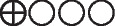 VERY LOW	CRITICAL
								Ethanol: 0% [358]	No reduction with ECTR		
								No antidote: Expected high	Probable reduction with ECTR		
**Irreversible neurological damage**											
Early EG poisoning *n* = 2 ^a^	Observational studies	Very serious ^b^	Not serious	Serious ^c^	Serious ^d^	Publication bias strongly suspected ^e^	This systematic review: 0%	Fomepizole: 0% [358]	No reduction with ECTR	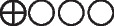 VERY LOW	CRITICAL
								Ethanol: 0% [358]	No reduction with ECTR		
								No antidote: Expected high	Probable reduction with ECTR		
**AKI requiring short-term dialysis**											
Early EG poisoning *n* = 2 ^a^	Observational studies	Very serious ^b^	Not serious	Serious ^c^	Serious ^d^	Publication bias strongly suspected ^e^	This systematic review: 15.5%	Fomepizole: 0.0% [358]	No reduction with ECTR	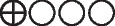 VERY LOW	CRITICAL
								Ethanol alone: 3.8% [358]	No reduction with ECTR		
								No antidote: Expected high	Probable reduction with ECTR		
**Length of ICU stay**											
Early EG poisoning *n* = 1 ^f^	Observational studies	Very serious ^b^	Not serious	Serious ^c^	Serious ^d^	Publication bias strongly suspected ^e^	This systematic review: Median 5 days [2, 8]	Fomepizole: Theoretically, ICU admission not needed	No reduction with ECTR	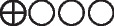 VERY LOW	IMPORTANT
								Ethanol: requires ICU admission and this would be prolonged	Data incomplete		
								No antidote: Expected high	Probable reduction with ECTR		
**Length of hospital stay**											
Early EG poisoning *n* = 2 ^a^	Observational studies	Very serious^b^	Not serious	Serious ^c^	Serious ^d^	Publication bias strongly suspected ^e^	This systematic review: Median 8 days [2, 15]	Fomepizole: Median 4 days [3, 5] [358]	No reduction with ECTR	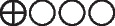 VERY LOW	IMPORTANT
								Ethanol: Median 3 days [2,4] [358]	No reduction with ECTR		
								No antidote: Expected high	Probable reduction with ECTR		
**Cost**											
Early EG poisoning *n* = 8^ g^	Observational studies	Very serious^b^	Not serious	Serious ^c^	Serious ^d^	Publication bias strongly suspected ^e^	Fomepizole: varies (See Table 6) [107–111, 227, 355, 427]	Fomepizole: varies (See Table 6) [107–111, 227, 355, 427]	Analyses and/or reporting incomplete. Cost of ECTR and fomepizole vary across countries and across institutions. HD appears cost-effective in smaller patients and high EG concentration	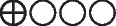 VERY LOW	IMPORTANT
							Ethanol: data incomplete (see Table 6) [227, 355]	Ethanol: No data	Data incomplete		
							No antidote: No data	No antidote: No data	No data		
**Serious complications of catheter insertion **^**h**^											
*n* = 5 ^i^	Observational studies	Not serious	Not serious ^j^	Not serious ^k^	Not serious ^l^	Strong association ^m^	Rate of serious complications of catheter insertion varies from 0.1% to 2.1%	≈ 0	Absolute effect is estimated to be varying from **1 to 21 more serious complications per 1000 patients** in the ECTR group	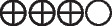 MODERATE	CRITICAL
**Serious complications of ECTR **^***n***^											
*n* = 6 ^o^	Observational studies	Not serious	Not serious	Not serious	Not serious	Strong association ^p^	Rate of serious complications of ECTR varies according to the type of ECTR performed from 0.005% (HD and CKRT), to 1.9% (HP)	≈ 0	Absolute effect is estimated to be varying from > **0 to 19 more serious complications per 1000 patients** in the ECTR group depending on the type of ECTR performed	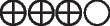 MODERATE	CRITICAL
**Complications related to antidote**											
*n* = 7 ^q^	Observational studies	Very serious^b^	Not serious	Serious ^c^	Serious ^d^	Publication bias strongly suspected ^e^	Fomepizole: Rare cases of anaphylaxis, bradycardia, hypotension [68, 428–432]		Smaller incidence with ECTR but minimal impact	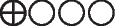 VERY LOW	IMPORTANT
							Ethanol: Altered consciousness in 5–15%, occasionally requiring mechanical ventilation [68, 431, 432], hypoglycemia in 16% of children [433], bradycardia in 10–12% [431, 432]		Smaller incidence with ECTR because of shorter duration of ethanol and lower risk of hypoglycemia because of dextrose in dialysate bath		

Bold text was inserted to highlight the result

*ECTR* Extracorporeal treatments, *HD* Hemodialysis, *CKRT* Continuous kidney replacement therapy, *HP* Hemoperfusion, *US* Ultrasound, *ICU* Intensive care unit

^#^Refers to patients with a glycolate concentration ≤ 12 mmol/L or an anion gap with potassium ≤ 28 mmol/L. As mentioned, an evidence profile table could not be constructed for patients with “late” ethylene glycol poisoning, i.e., those with a serum glycolate over 12 mmol/L, or an anion gap over 28 mmol/L, although ECTR would be considered lifesaving in this context

^*^ Cases of ECTR were regrouped regardless of antidote used

a. Includes our systematic review of the literature on ECTR (181 case reports and 446 patients, 84 of which had “Early EG poisoning”) and 1 systematic review on standard care alone

b. Case reports published on effect of ECTR. Uncontrolled and unadjusted for confounders such as severity of poisoning, co-ingestions, supportive and standard care, and co-interventions. Confounding-by-indication is inevitable since ECTR was usually attempted when other therapies have failed

c. ECTR and standard care performed may not be generalizable to current practice (literature pre-dating 2000)

d. Few events in small sample size, optimal information size criteria not met

e. Publication bias is strongly suspected due to the study design (case reports published in toxicology)

f. Includes our systematic review of the literature on ECTR 181 case reports (446 patients, 84 of which had “Early EG poisoning”)

g. Includes 8 articles

h. For venous catheter insertion: serious complications include hemothorax, pneumothorax, hemomediastinum, hydromediastinum, hydrothorax, subcutaneous emphysema retroperitoneal hemorrhage, embolism, nerve injury, arteriovenous fistula, tamponade, and death. Hematoma and arterial puncture were judged not serious and thus excluded from this composite outcome. DVT and infection complications were not included considering the short duration of catheter use

i. Based on 5 single-arm observational studies: 2 meta-analyses comparing serious mechanical complications associated with catheterization using or not an ultrasound, which included 6 RCTs in subclavian veins [434] and 11 in internal jugular veins [435]; 2 RCTs comparing major mechanical complications of different sites of catheterization [436, 437]; and one large multicenter cohort study reporting all mechanical complications associated with catheterization [438]. Rare events were reported from case series and case reports

j. Not rated down for inconsistency since heterogeneity was mainly explained by variation in site of insertion, use of ultrasound, experience of the operator, populations (adults and pediatric), urgency of catheter insertion, practice patterns and methodological quality of studies

k. Not rated down for indirectness since cannulation and catheter insertion was judged similar to the procedure for other indications

l. Not rated down for imprecision since wide range reported explained by inconsistency

m. The events in the control group are assumed to be zero (since no catheter is installed for ECTR), therefore, the magnitude of effect is at least expected to be large, which increases the confidence in the estimate of effect. Furthermore, none of the studies reported 95%CI which included the null value and all observed complications occurred in a very short time frame (i.e., few hours)

n. For HD and CKRT: serious complications (air emboli, shock and death) are exceedingly rare especially if no net ultrafiltration. Minor bleeding from heparin, transient hypotension, and electrolytes imbalance were judged not serious. For HP: serious complications include severe thrombocytopenia, major bleeding, and hemolysis. Transient hypotension, hypoglycemia, hypocalcemia, and thrombocytopenia were judged not serious

o. HD/CKRT: Based on 2 single-arm studies describing severe adverse events per 1000 treatments in large cohorts of patients [439, 440]. HP: Based on 2 small single-arm studies in poisoned patients [441, 442]. Rare events were reported in case series and case reports

p. Assuming that patients in the control group would not receive any form of ECTR, the events in the control group would be zero; therefore, the magnitude of effect is at least expected to be large, which increases the confidence in the estimate of effect. Furthermore, none of the studies reported 95%CI which included the null value and all observed complications occurred in a very short time frame (i.e., few hours)

q. Includes seven observational cohorts of patients receiving ethanol or fomepizole

The evidence table was therefore populated with the subset of patients known to have a good prognosis without ECTR, i.e., those with a glycolate concentration ≤ 12 mmol/L or an anion gap with potassium ≤ 28 mmol/L (“Early EG poisoning,” Table 7) [29, 66, 71, 358]. As expected, the addition of ECTR to fomepizole did not seem to improve the prognosis of these patients, namely mortality, length of stay, or requirement of ECTR for AKI. ECTR appears to reduce costs, although this must be weighed against possible risks of complications of ECTR. When ethanol is used in low-risk patients, ECTR reduces the duration of time during which a patient is exposed to risks related to ethanol (decreased consciousness, dysphoria) and may limit the risk of ethanol failure. In rare cases in which neither fomepizole nor ethanol can be used (a situation described by some panel members), ECTR would theoretically markedly reduce the risk of mortality and AKI.

### Clinical recommendations

The EXTRIP recommendations for ECTR in EG poisoning are presented in Table 8. Current indirect evidence suggests that ECTR is lifesaving when significant acidemia and/or Stage 2 or 3 AKI are present as it corrects metabolic disequilibria and removes EG, oxalate, and glycolate. EG-related mortality increases substantially once the plasma glycolate concentration exceeds 12 mmol/L [71]. Uncertainty remains on the expected magnitude of effect of ECTR compared to no ECTR on mortality and other patient-important outcomes at different plasma glycolate concentrations. When neither AKI nor acidemia is present, the advantages of ECTR when added to an ADH inhibitor are mainly to limit costs, reduce length of hospitalization, and limit risks of ethanol therapy when used, rather than reducing the occurrence of major adverse outcomes from EG. These recommendations may be used to prioritize and triage ECTR in cases of group poisoning [24, 359]. Because of the lack of reliable comparative studies and the aforementioned uncertainties, the quality of evidence was very low for all recommendations.

**Table 8:** Final EXTRIP recommendations on the use of ECTR in ethylene glycol poisoning

INDICATIONS* EG Dose a. In patients presenting with EG poisoning, **we recommend against** ECTR based solely on the reported EG dose Plasma EG concentration a. Fomepizole is used i. In patients presenting with EG poisoning, **we suggest** ECTR if EG concentration is > 50 mmol/L (> 310 mg/dL) b. Ethanol is used i. In patients presenting with EG poisoning, **we recommend** ECTR if EG concentration is > 50 mmol/L (> 310 mg/dL) ii. In patients presenting with EG poisoning, **we suggest** ECTR if EG concentration is 20–50 mmol/L (124–310 mg/dL) c. No antidote is available i. In patients presenting with EG poisoning, **we recommend** ECTR if EG concentration is > 10 mmol/L (> 62 mg/dL) Osmol gap (calculated as measured osmolality − calculated osmolarity, in SI units and adjusted for ethanol) when there is evidence of EG exposure a. Fomepizole is used i. In patients presenting with EG poisoning, **we suggest** ECTR if the osmol gap is > 50 b. Ethanol is used i. In patients presenting with EG poisoning, **we recommend** ECTR if the osmol gap is > 50 ii. In patients presenting with EG poisoning, **we suggest** ECTR if the osmol gap is 20–50 c. No antidote is available i. In patients presenting with EG poisoning, **we recommend** ECTR if the osmol gap is > 10 Plasma glycolate concentration a. In patients presenting with EG poisoning, **we recommend** ECTR if the glycolate concentration is > 12 mmol/L b. In patients presenting with EG poisoning, **we suggest** ECTR if the glycolate concentration is 8–12 mmol/L Anion gap (calculated as Na^+^ + K^+^ − Cl^−^ − HCO_3_^−^) when there is evidence of EG exposure a. In patients presenting with EG poisoning, **we recommend** ECTR if the anion gap is > 27 mmol/L b. In patients presenting with EG poisoning, **we suggest** ECTR if the anion gap is 23–27 mmol/L Clinical indications a. Coma i. In patients presenting with coma due to EG poisoning, **we recommend** ECTR b. Seizures i. In patients presenting with EG poisoning and seizures, **we recommend** ECTR c. Kidney Impairment i. In patients presenting with EG poisoning and CKD (eGFR < 45 mL/min/1.73m^2^), **we suggest** ECTR ii. In patients presenting with EG poisoning and AKI (KDIGO stage 2 or 3), **we recommend** ECTR **MODALITY** a. In patients presenting with EG poisoning requiring ECTR, when all ECTR modalities are available, **we recommend** using intermittent hemodialysis rather than any other type of ECTR b. In patients presenting with EG poisoning requiring ECTR, **we recommend** using continuous kidney replacement therapy over other types of ECTR if intermittent hemodialysis is not available **CESSATION** a. **We recommend** stopping ECTR when the anion gap (calculated as Na^+^ + K^+^ − Cl^−^ − HCO_3_^−^) is < 18 mmol/L b. **We suggest** stopping ECTR when the EG concentration is < 4 mmol/L (25 mg/dL) c. **We suggest** stopping ECTR when acid–base abnormalities are corrected	**1**

Bold text was inserted to highlight the result

*ECTR* extracorporeal treatment, *EG* Ethylene glycol, *CKD* Chronic kidney disease, *AKI* Acute kidney injury, *KDIGO* Kidney Disease Improving Global Outcomes

^*^If any of indication criteria fulfills a recommendation for ECTR, then ECTR should be performed regardless of the presence of other conditions

#### Indications

The following indications should be considered independent of each other. For example, if a criterion recommending ECTR is met, then evaluation of the other criteria is not necessary.

##### EG Dose

- In patients presenting with EG poisoning, **we recommend against** ECTR based solely on the reported EG dose ingested (strong recommendation, very low-quality evidence)

Rationale: A reported EG dose ingested is never by itself an indication for ECTR as it may be imprecise and requires complementary confirmation from other diagnostic cues such as the presence of EG in blood, an elevated osmol gap and/or an elevated anion gap, or other non-specific tests (calcium oxalate crystals in urine). However, a history of ingestion may prompt early contact and consideration of transfer to a center where ECTR can be performed should this be required. Similarly, neither oxalate crystals in urine, urine immunofluorescence nor hypocalcemia nor a history of EG exposure alone are indications for ECTR (but may help to diagnose EG poisoning).

##### Plasma EG concentration

- Fomepizole is used:
  i. In patients presenting with EG poisoning, **we suggest** ECTR if EG concentration is > 50 mmol/L (> 310 mg/dL) (weak recommendation, very low-quality evidence)
- Ethanol is used
  i. In patients presenting with EG poisoning, **we recommend** ECTR if EG concentration is > 50 mmol/L (> 310 mg/dL) (strong recommendation, very low-quality evidence)
  ii. In patients presenting with EG poisoning, **we suggest** ECTR if EG concentration is 20 to 50 mmol/L (124 to 310 mg/dL) (weak recommendation, very low-quality evidence)
- No antidote is available
  i. In patients presenting with EG poisoning, **we recommend** ECTR if EG concentration is > 10 mmol/L (> 62 mg/dL) (strong recommendation, very low-quality evidence)

Rationale: The EG concentration is poorly prognostic of clinical outcomes because EG itself causes little toxicity. In fact, patients receiving fomepizole alone have excellent outcomes regardless of the EG concentration, assuming there is no kidney impairment and minimal academia [358]. The benefit of ECTR in this context is to presumably reduce length of stay and total hospital costs, especially at high EG concentration, rather than reducing the incidence of outcomes such as mortality or AKI and explains why this is "suggested" rather than "recommended" [107–111, 227, 267]. However, the workgroup acknowledges that these decisions need to be individualized (which GRADE emphasizes for weak/conditional recommendations) as cost considerations are dependent on the setting and institution; for example, costs for ECTR and fomepizole may exceed fomepizole alone if a patient needs to be transferred to another center for ECTR.

The same EG concentration cutoff was chosen when ethanol was used as an antidote, although this was a recommendation. The rationale being that ADH blockade with ethanol is more unpredictable and there are cases of treatment failure even with little to no acidosis or kidney impairment present on admission [358]. Prolonged ethanol therapy also carries risks and requires admission to a high-dependency unit which may be shortened by using ECTR. With EG concentration > 50 mmol/L, assuming an endogenous EG T_1/2_ = 14 h during ethanol therapy, a patient would need treatment for > 48 h before reaching a safe EG concentration, during which the risks of side effects from ethanol (central nervous system depression, dysphoria) and/or therapeutic failure become considerable. If no ADH inhibitor can be used, an EG concentration > 10 mmol/L (> 62 mg/dL) should prompt ECTR, as adverse outcomes are generally reported in untreated patients over this concentration; however, until there is a better understanding of threshold ethylene glycol concentrations, the workgroup recognizes that a more cautious cutoff may be preferable (see research gap below). From a known EG concentration and using local cost of fomepizole, ethanol, hospitalization, and ECTR, and using the EG T_1/2_ during specific circumstances (ECTR, AKI, antidote used, Table 1 and Table 2), clinicians can estimate the time to reach a safe concentration and decide if ECTR would be cost-effective in the specific scenario (Additional file 1: Figure S3). The decision to transfer a patient to another institution to receive ECTR should be individualized.

At extremely high EG concentrations with an associated high plasma osmolality, there is a potential risk of inducing osmotic disequilibrium with ECTR. However, these complications are considered unlikely because of the acute onset of the hyperosmolality and were only reported in one out of the 27 cases with an EG concentration > 100 mmol/L(> 620 mg/dL) [268]. The workgroup’s recommendations remain applicable for these patients.

##### The osmol gap (calculated as measured osmolality − calculated osmolarity, in SI units and adjusted for ethanol) when there is evidence of EG exposure

- Fomepizole is used
  i. In patients presenting with EG poisoning, **we suggest** ECTR if the osmol gap is > 50 (weak recommendation, very low-quality evidence)
- Ethanol is used
  i. In patients presenting with EG poisoning, **we recommend** ECTR if the osmol gap is > 50 (strong recommendation, very low-quality evidence)
  ii. In patients presenting with EG poisoning, **we suggest** ECTR if the osmol gap is 20 to 50 (weak recommendation, very low-quality evidence)
- No antidote is available
  i. In patients presenting with EG poisoning, **we recommend** ECTR if the osmol gap is > 10 (strong recommendation, very low-quality evidence)

Rationale: EG assays are seldom available locally in an appropriate time frame to influence clinical decisions [360, 361], and so the osmol gap is often used as a surrogate to predict the EG concentration [113, 119, 172, 342, 362–364]. Unfortunately, many clinical conditions and ingestion of other alcohols increase the osmol gap [365]. Conversely, the osmol gap may be “normal” or even below 0 if EG is already metabolized or if too little EG is ingested [24, 366–369]. For these reasons, the osmol gap is a poor screening test for EG ingestion, especially at low osmol gap values [370–373]. However, at high EG concentration, the osmol gap correlates linearly with the EG concentration (Additional file 1: Table S11), despite considerable inter- and intra-patient variability [371]. The panel proposed that the same cutoffs for osmol gap and EG concentrations be used for initiation of ECTR, especially if there is a confirmed history of EG ingestion. Since the osmol gap may overestimate the EG concentration, the panel acknowledges that using these cutoffs may lead to unnecessary ECTRs. If no antidote is available, an osmol gap > 10, in the context of EG exposure, is a reasonable criterion for hemodialysis, with the above caveats [371]. The workgroup did not provide an osmol gap cutoff when there is no/very low suspicion of EG poisoning. The workgroup also acknowledges that there are many formulas to calculate to osmol gap [368, 369, 374, 375].

##### Plasma glycolate concentration

- In patients presenting with EG poisoning, **we recommend** ECTR if the glycolate concentration is > 12 mmol/L (strong recommendation, very low-quality evidence)
- In patients presenting with EG poisoning, **we suggest** ECTR if the glycolate concentration is > 8 mmol/L (weak recommendation, very low-quality evidence)

Rationale: Glycolate is the EG metabolite in highest concentration in blood [19] and correlates with AKI and death [71]. There was only one death reported when the glycolate concentration was < 12 mmol/L [376]; however, the mortality rate rises substantially once the glycolate concentration exceeds 12 mmol/L [71].

##### Anion gap when there is evidence of EG exposure

- In patients presenting with EG poisoning, **we recommend** ECTR if the anion gap (calculated as Na^+^ + K^+^– Cl^−^– HCO_3_.^−^) is > 27 mmol/L (strong recommendation, very low-quality evidence)
- In patients presenting with EG poisoning, **we suggest** ECTR if the anion gap is 23–27 mmol/L (weak recommendation, very low-quality evidence)

Rationale: Glycolate assays are not available in most institutions. The anion gap is by far the best surrogate marker for glycolate and correlates linearly with glycolate and is associated with clinical outcomes [29, 67, 71, 72, 74]. Based on previous reviews [71], the workgroup agreed that an anion gap of 24–28 mmol/L and > 28 mmol/L, respectively, would best correlate with the suggested and recommended indications for ECTR based on plasma glycolate concentrations. To support this, there were only 3 patients out of 84 with an anion gap ≤ 28 mmol/L or glycolate concentration ≤ 12 mmol/L on admission who died; in all these cases, the clinical and metabolic data were incomplete (Additional file 1: Table S10). Because the relationship between glycolate concentration and prognosis require confirmation, the workgroup chose slightly more conservative cutoffs, accepting that this may lead to unnecessary ECTRs.

The anion gap may overestimate (e.g., concomitant AKI or ketoacidosis) or underestimate (e.g., hypoalbuminemia or co-ingestions of lithium or barium) [377–379] the glycolate concentration. In such circumstances, other acid–base parameters such as pH, HCO_3_^−^, and base excess can also be consulted with due consideration of the extent to which they are also influenced by other factors such as inadequate respiratory compensation or exogenous bicarbonate. It is important to note that the anion gap is only useful to predict glycolate concentrations if there is a high pre-test probability of EG exposure. Its value to predict the need for ECTR is poor if it is used indiscriminately; when there is little or no evidence of EG exposure, an elevated anion gap is not by itself an indication for ECTR as this may be caused by various factors.

An elevated glycolate concentration can falsely elevate the plasma lactate concentration on some analyzers [239, 380–384], which has prompted some to suggest using the “lactate gap” as a surrogate of glycolate. However, this requires knowledge of the specific analyzer’s cross-reactivity so it cannot be simply formalized into a recommendation.

Some authors suggest that hemodialysis may be obviated even in cases of severe academia [385] or that fomepizole may lower glycolate concentrations faster than hemodialysis [124], although the EXTRIP panel strongly cautions against these viewpoints.

##### Clinical indications

- Coma
  i. In patients presenting with coma due to EG poisoning, **we recommend** ECTR (strong recommendation, very low-quality evidence)
- Seizures
  i. In patients presenting with EG poisoning and seizures, **we recommend** ECTR (strong recommendation, very low-quality evidence)
- Kidney Impairment
  i. In patients presenting with EG poisoning and CKD (eGFR < 45 mL/min/1.73 m.^2^), **we suggest** ECTR (weak recommendation, very low-quality evidence)
  ii. In patients presenting with EG poisoning and AKI (KDIGO stage 2 or 3), **we recommend** ECTR (strong recommendation, very low-quality evidence)

Rationale: AKI is correlated with mortality [27, 29] and kidney impairment reduces endogenous elimination of EG, which is pertinent once antidotal therapy is started, as elimination of EG and metabolites are dependent on functional kidneys. The presence of kidney impairment lowers the EG concentration thresholds for initiating ECTR. Coma and seizures are associated with a poor prognosis and become a clinical justification for ECTR [23, 27, 31, 32, 63, 65, 67, 74]. Mild inebriation may be due to EG early after ingestion and is not an indication for ECTR.

Other clinical manifestations are not recommendations for ECTR; respiratory failure and pulmonary edema would occur after already stated indications for ECTR. Cranial nerve defects may occur long after exposure despite repeated ECTR.

##### Special populations

The workgroup proposed that these recommendations remain applicable for other populations. A lower EG threshold concentration for ECTR may be applicable in children being treated with ethanol, to minimize adverse effects of ethanol. In pregnancy, ethanol is potentially teratogenic and fomepizole is classed Category C [386]. Although fomepizole was used without complication in the second [387] and third trimesters [388], there may be a preference for lower ECTR threshold to reduce the exposure to these antidotes in the pregnant patient, although ECTR also carries risks in this population. These considerations were discussed with a patient representative who endorsed the recommendations.

#### Modality

- In patients presenting with EG poisoning requiring ECTR, when all ECTR modalities are available, **we recommend** using intermittent hemodialysis rather than any other type of ECTR (strong recommendation, very low-quality evidence)
- In patients presenting with EG poisoning requiring ECTR, **we recommend** using continuous kidney replacement therapy (CKRT) over other types of ECTR if intermittent hemodialysis is not available (strong recommendation, very low-quality evidence)

Rationale: Hemodialysis remains the ECTR that is most widely available so can be initiated quicker than other ECTRs and is also less costly [389]. High-efficiency hemodialysis is also the most efficient ECTR to remove both EG and metabolites, although intermittent hemodiafiltration would be expected to be equally effective. Intermittent hemodialysis was also shown, in methanol poisoning, to correct acidemia quicker than CKRT [390], although clinical outcomes were comparable with both techniques [391]. CKRT is preferred if it can be initiated faster than hemodialysis (e.g., unavailability of hemodialysis, nursing limitations), leading to attainment of a safe EG concentration, as illustrated in Additional file 1: Figure S3. CKRT is preferable if a patient has marked brain edema, as it increases intracranial pressure to a lesser degree than intermittent hemodialysis [392]. Clinicians performing ECTR should optimize operator settings to maximize EG clearance (e.g., higher blood flow, higher effluent production, filters with higher surface area).

In resource-restricted regions where hemodialysis or CKRT cannot be performed, rapid-exchange peritoneal dialysis using non-lactate-based solutions will at least double EG clearance if kidney impairment is present. However, peritoneal dialysis should not replace hemodialysis if the latter is available, as there are many cases of clinical worsening during peritoneal dialysis [150, 158, 393]. Although there are authors suggesting adding peritoneal dialysis to hemodialysis, the EXTRIP panel could not imagine a clinical or kinetic rationale for this, as peritoneal dialysis would only add 5% to the clearance obtained with hemodialysis [52] but at much higher cost and complication rate.

#### Cessation

- **We recommend** stopping ECTR when the AG (calculated as Na^+^ + K^+^– Cl^−^– HCO_3_.^−^) is < 18 mmol/L (strong recommendation, very low-quality evidence)
- **We suggest** stopping ECTR when the EG is < 4 mmol/L (25 mg/dL) (weak recommendation, very low-quality evidence)
- **We suggest** stopping ECTR when acid–base abnormalities are corrected (weak recommendation, very low-quality evidence)

Rationale: Once ECTR is initiated, it is recommended that all acid–base parameters have normalized, in particular a confirmed and sustained normalization of the glycolate concentration and/or anion gap before stopping ECTR. Some clinical markers such as seizures may reverse quickly once ECTR is initiated and others, such as cranial nerve palsies and AKI in particular, may take weeks to resolve and should therefore not guide cessation. Once acid–base homeostasis is restored, then an acceptable and conservative endpoint for cessation is an EG concentration < 4 mmol/L. If ECTR must be terminated when the EG concentration is higher (e.g., multiple poisoned patients), continuation of ADH blockade is reasonable to prevent further EG metabolism. As noted above, some authors have suggested that an EG concentration < 10 mmol/L is an acceptable endpoint in asymptomatic patients [60], but supporting clinical data are not currently available and there are few downsides to continuing ECTR until lower cutoffs are reached. The clinical significance of rebound in EG concentration after ECTR is uncertain but can be addressed with a repeat ECTR session if clinically indicated. Although the EG concentration is linearly correlated with the osmol gap when elevated, the workgroup did not propose a specific osmol gap cutoff for cessation as there are too many imprecisions at low osmol gap values. The use of validated equations based on expected decay of EG in plasma can be used to predict dialysis time [67, 115, 233]. These formulas assume high-efficiency hemodialysis, with good and constant blood flows, and no sampling errors [394]. These formulas may be useful for planning purposes, especially in cases of multiple poisoned patients where ECTR triage is required [67], although the workgroup reiterates that all acid–base abnormalities should be reversed before stopping ECTR.

#### Miscellaneous

There are various reported approaches to ADH blockade once ECTR is started. Some centers switch from fomepizole to ethanol and others use ethanol in the dialysate bath. Both fomepizole and ethanol are readily dialyzable (see above) and require increased dosage during ECTR (Additional file 1: Table S12). Some centers withhold ADH blockade during ECTR, claiming that ECTR will remove metabolites quickly enough to avoid harm [395]. This appeared to be safe in 5 patients in a retrospective study [123], but more data are required and this approach cannot be currently recommended.

Several EG-poisoned patients have concomitant alcohol use disorder, and are at risk for alcohol withdrawal, especially if ECTR is performed [282]; usual measures should be in place to mitigate this risk. Cerebral hemorrhage is not a common manifestation of EG poisoning (as opposed to methanol) but occasionally occurs [396, 397], and so the decision to anticoagulate the ECTR circuit should be individualized.

EXTRIP strongly advocates for widespread and rapid (2–4 h) hospital availability of glycolate and EG measurements, as recommended by the National Academy of Clinical Biochemistry [398]. These would permit precise diagnosis and targeted treatment, avoiding costly unnecessary treatments [399, 400].

## Research gap

Cost and clinical outcome studies should be performed to evaluate the use of fomepizole versus ethanol versus no ADH blockade during ECTR. Further, in patients with no acidosis, it would be useful to determine if the addition of ECTR to ADH blockade significantly reduces cost and/or length of stay. These questions can be ideally answered by prospective randomized trials, but adequately powered studies require multicenter collaborations which can be complicated. Well-conducted single-center studies with complete reporting of relevant clinical outcomes including biomarkers of EG toxicity (for example, changes in acidosis and kidney function), preferably with statistical matching of baseline variables, will also be informative. Of course, cost-effectiveness studies will vary between institutions and health systems, so the development of algorithms that allow for the incorporation of all relevant components and their cost to facilitate local decision-making is anticipated to be informative. Studies should identify what represents “safe” EG and glycolate concentrations to better define initiation and cessation criteria for ECTR and/or ADH blockade. Cases of osmotic demyelination syndrome in those that have a very high concentration of EG (e.g., > 100 mmol/L or > 620 mg/dL) and of “dialysis disequilibrium” during ECTR should be reported to assess the incidence of these phenomena [268, 401]. Cases of EG poisoning treated with exchange transfusion (when no other modality is available) should be reported with proper toxicokinetic data.

## Conclusion

The EXTRIP workgroup reviewed the available literature pertaining to the role of ECTR in ethylene glycol poisoning to propose treatment recommendations based on blood tests with and without the use of ADH blockers. The presence of kidney impairment decreased the thresholds for ECTR use. The workgroup recommends using intermittent hemodialysis over other ECTRs, and to evaluate the cost-benefit between fomepizole use vs ECTR for less severe poisoning. The dosage of antidotes needs to be adjusted during ECTR. The workgroup strongly advocates for the widespread availability of resources for the rapid measurement of glycolate and EG concentrations.

## Supplementary Information

**Additional file 1**. Supplemental file.

## Data Availability

The data underlying this article will be shared on reasonable request to the corresponding author.
