# Supplementary material for: Extracorporeal treatment for ethylene glycol poisoning: systematic review and recommendations from the EXTRIP workgroup
Source: Crit Care. 2023 Feb 10;27:56. doi: 10.1186/s13054-022-04227-2 (PMC9921105; doi:10.1186/s13054-022-04227-2)
Supplement: Supplementary file 1 — Additional file 1. Supplemental file. [file 13054_2022_4227_MOESM1_ESM.pdf]

SUPPLEMENTAL FILE

EXTRIP CLINICAL PRACTICE GUIDELINE

As defined by the Institute of Medicine in 2011, clinical practice guidelines are statements that include recommendations intended to optimize patient care by assisting practitioners and patients in making decisions about appropriate health care for specific clinical circumstances.[1] They are informed by a systematic review of evidence and an assessment of the benefits and harms of alternative care options. Attributes of good guidelines include validity, reliability, reproducibility, clinical applicability, clinical flexibility, clarity, multidisciplinary process, review of evidence, and documentation.

The EXTRIP-2 workgroup pursued a second phase of the international multidisciplinary effort started in 2010[2]. Using established methodology[3], the EXTRIP workgroup reviewed the literature and developed recommendations on the use of extracorporeal treatments (*ECTR*) in the context of poisoning for a new set of 10 poisons (*see glossary*). More specifically, the effect of ECTRs in addition to standard care was compared against standard care alone. Potential benefits of ECTRs were balanced with potential harms from the procedure. Outcomes measured included mortality, relevant clinical and physiological end points, complications associated with ECTR procedure, as well as the extent of extracorporeal removal of the poison (i.e., dialyzability, see glossary see below). When applicable, different populations (including end-stage kidney disease and pediatric patients), types of poisoning (acute, acute-on-chronic, and chronic), and types of ECTR were evaluated. When developing recommendations, variations in clinical practice in different settings across the globe were considered especially regarding resource use, costs, and availability of antidotes and ECTR.[4] Implementation issues were addressed when appropriate.

Workgroup composition

The EXTRIP workgroup is an international collaborative comprising recognized experts from various clinical specialties (medical toxicology, emergency medicine, nephrology, critical care, pediatrics, and pharmacology). The EXTRIP-2 workgroup renewed the participation of 15 members and invited 22 new experts to join the workgroup and increase representativeness across the world (Table S1). Six co-chairs (M.G., S.G., R.S.H., V.L., T.D.N., and D.M.R.), of which, one is a non-voting guideline methodologist (V.L.) led and supervised the different aspects of the guideline development.

Supplemental Table S1. Represented societies

African Federation of Emergency Medicine<br>Acute Dialysis Quality initiative<br>American College of Clinical Pharmacology<br>American Academy of Clinical Toxicology<br>American College of Emergency Physicians<br>American College of Medical Toxicology<br>American Society of Nephrology<br>American Society of Pediatric Nephrology<br>Asia Pacific Association of Medical Toxicology*<br>Asian Pacific Nephrology Association<br>Australian and New Zealand Intensive Care Society*<br>Australian and New Zealand Society of Nephrology*<br>Brazilian Association of Information Centres and Toxicologic Assistance*<br>Brazilian Society of Toxicology<br>Canadian Association of Emergency Physicians<br>Chinese College of Emergency Physicians<br>Chinese Medical Association	European Association of Poison Centres and Clinical Toxicologists<br>European Renal Association-European Dialysis and Transplant Association<br>European Society for Emergency Medicine<br>European Society of Intensive Care Medicine<br>Faculty of Intensive Care Medicine<br>International Pediatric Nephrology Association<br>International Society of Nephrology<br>Middle East and North Africa Clinical Toxicology Association<br>National Kidney Foundation<br>Pediatric Continuous Renal Replacement Therapy<br>Pediatric Critical Care Medicine<br>Society of Academic Emergency Medicine<br>Society of Critical Care Medicine<br>The Renal Association

*This representation does not signify endorsement. Recommendations will be submitted to societies for review and potential endorsement.

Disclosure and management of potential COI

All prospective members were required to disclose any actual, potential, or perceived COI prior to inclusion in the workgroup. The disclosures were used to categorize the members as cleared for full participation, allowed to participate with recusal from certain aspects of guideline development, or disqualified from participation. The co-chairs remained free of any financial COI during the entire guideline development process, meaning avoidance of interests and relationships with pharmaceutical or device companies pertaining to the topic of poisoning. Members were required to disclose to the co-chairs any new activities that had the potential to be viewed as a COI prior to engaging in the activity, at the beginning of face-to-face meeting, and before submission of the manuscript. Co-chairs determined if specific activities were allowed under the COI rules. All COIs deemed as potential appearance of a conflict of interest were required to be included in the manuscript.

Framing of the clinical question

An initial list of relevant clinical questions was developed for each poison, reviewed by the co-chairs, and then approved by the entire workgroup prior to the first iteration of EXTRIP-2. Clinical questions were formulated following the standard PICO format. Patient population and specific subpopulations (e.g., type of poisoning / exposure, patients with/without kidney impairment) as well as interventions of interest (e.g., type of ECTR) were defined for all clinical questions, as applicable. Comparator(s) of interest were explicitly defined as the standard care (with/without antidotes) for every poison (Table S2).

Supplemental Table S2: Standard care for each poison reviewed

Poison / drug	"Standard care"	Comparison
Early ethylene glycol*	Standard 1: ABC, decontamination, bicarbonate, pyridoxine/thiamine, ethanol	Standard 1 vs Standard 1 + ECTR
	Standard 2: ABC, decontamination, bicarbonate, pyridoxine/thiamine, fomepizole	Standard 2 vs Standard 2 + ECTR

**Outcomes were only compared for patients with early EG poisoning, i.e., those with a glycolate concentration <12mmol/L or an anion gap <28mmol/L. In other groups, no proper comparison could be made (lack of controls, no clinical equipoise), and ECTR was considered lifesaving.*

Selection and ranking of patient-important outcomes

All outcomes of interest were identified *a priori,* and the workgroup members explicitly rated their relative importance for decision making. In general, patient-important outcomes considered were: mortality, serious or permanent end-organ injury specific to each poison reviewed, serious complications of catheter insertion (see glossary), serious complications of ECTR (see glossary), length of ICU stay, length of hospital stay, and costs. Ranking of the outcomes by importance for decision-making (either "critical", 'important but not critical" or "of limited importance") was determined by consensus for each poison reviewed and for PICO question developed.

Dialyzability was reported as a separate outcome: the workgroup acknowledged that excellent poison removal does not correlate with clinical improvement for certain poisons. Conversely, clinical improvement can sometimes be observed despite poor extracorporeal removability.

Systematic review: balance of benefits and harms of ECTR in ethylene glycol poisoning

The objectives of this systematic review of the literature were to: 1) summarize the balance of benefits and harms of ECTR on patient important outcomes, and 2) describe the toxicokinetic outcomes of ECTR in the context of severe toxicity to ethylene glycol.

One health sciences librarian (E.G.) in collaboration with one co-chair (M.G) and the methodologist (V.L.) designed literature searches to address clinical questions. PubMed / MEDLINE, and EMBASE were searched initially for prior systematic reviews that have addressed similar topics. Based on the result of this initial landscape assessment of the literature and based on *a priori* knowledge of the literature, an assumption was made by the workgroup that no or very few studies directly comparing the effect of ECTR to standard care would be identified. Thus, the initial search strategy was designed to systematically search and identify all studies

describing the effect of ECTR on patient important outcomes or toxicokinetic outcomes. If the initial search of the literature did not identify comparative studies in which ECTR was measured against standard care alone (with or without an antidote, if applicable), complementary searches were designed to systematically search and identify studies describing patient important outcomes and prognostic factors in patients receiving the standard care alone. In addition to the mentioned systematic reviews, a complementary search was designed to identify the best available evidence describing harms associated with catheter insertion and ECTR procedure. The systematic review protocol is reported in accordance with the PRISMA-P 2015 Checklist (Table S3), as appropriate.

Supplemental Table S3: PRISMA-P 2015 checklist [5]

Section and topic			#	Checklist item	Information reported
**ADMINISTRATIVE INFORMATION**	**Title**	Identification	1a	Identify the report as a protocol of a systematic review	**Y**
		Update	1b	If the protocol is for an update of a previous systematic review, identify as such	**N/A**
	**Registration**		2	If registered, provide the name of the registry (e.g., PROSPERO) and registration number	**N**
	**Authors**	Contact	3a	Provide name, institutional affiliation, and e-mail address of all protocol authors; provide physical mailing address of corresponding author	**Y**
		Contributions	3b	Describe contributions of protocol authors and identify the guarantor of the review	**Y**
	**Amendments**		4	If the protocol represents an amendment of a previously completed or published protocol, identify as such and list changes; otherwise, state plan for documenting important protocol amendments	**N/A**
	**Support**	Sources	5a	Indicate sources of financial or other support for the review	**Y**
		Sponsor	5b	Provide name for the review funder and/or sponsor	**Y**
		Role of sponsor or funder	5c	Describe roles of funder(s), sponsor(s), and/or institution(s), if any, in developing the protocol	**Y**
**INTRODUCTION**	**Rationale**		6	Describe the rationale for the review in the context of what is already known	**Y**
	**Objectives**		7	Provide an explicit statement of the question(s) the review will address with reference to participants, interventions, comparators, and outcomes (PICO)	**Y**
**METHODS**	**Eligibility criteria**		8	Specify the study characteristics (such as PICO, study design, setting, time frame) and report characteristics (such as years considered, language, publication status) to be used as criteria for eligibility for the review	**Y**
	**Information sources**		9	Describe all intended information sources (such as electronic databases, contact with study authors, trial registers or other grey literature sources) with planned dates of coverage	**Y**
	**Search strategy**		10	Present draft of search strategy to be used for at least one electronic database, including planned limits, such that it could be repeated	**Y**
	**Study records**	Data management	11a	Describe the mechanism(s) that will be used to manage records and data throughout the review	**Y**
		Selection process	11b	State the process that will be used for selecting studies (such as two independent reviewers) through each phase of the review (that is, screening, eligibility, and inclusion in meta-analysis)	**Y**
		Data collection process	11c	Describe planned method of extracting data from reports (e.g., piloting forms, done independently, in duplicate), any processes for obtaining and confirming data from investigators	**Y**
	**Data items**		12	List and define all variables for which data will be sought (such as PICO items, funding sources), any pre-planned data assumptions and simplifications	**Y**
	**Outcomes and prioritization**		13	List and define all outcomes for which data will be sought, including prioritization of main and additional outcomes, with rationale	**Y**
	**Risk of bias in individual studies**		14	Describe anticipated methods for assessing risk of bias of individual studies, including whether this will be done at the outcome or study level, or both; state how this information will be used in data synthesis	**Y**
	**Synthesis**		15a	Describe criteria under which study data will be quantitatively synthesized	**Y**
			15b	If data are appropriate for quantitative synthesis, describe planned summary measures, methods of handling data, and methods of combining data from studies, including any planned exploration of consistency (such as I^2, Kendall's tau)	**Y**
			15c	Describe any proposed additional analyses (e.g., sensitivity or subgroup analyses, meta-regression)	**Y**
			15d	If quantitative synthesis is not appropriate, describe the type of summary planned	**N/A**
	**Meta-bias(es)**		16	Specify any planned assessment of meta-bias(es) (such as publication bias across studies, selective reporting within studies)	**N/A**
	**Confidence in cumulative evidence**		17	Describe how the strength of the body of evidence will be assessed (such as GRADE)	**Y**

Legend: Y; yes, N; no, N/A; not applicable

Literature search: Effect of ECTR

Eligibility criteria: as per EXTRIP initial methodology [3], eligibility criteria for study selection were based on:

-Types of study design: all types, including randomized controlled trials, non-randomized comparative studies, one-arm studies, case series/case reports, abstracts from scientific and clinical meetings. Modeling studies, animal experiments and *in vitro* experiments are accepted if the methods and results are interpretable and correlated in humans.

-Type of participants: all patients with severe poisoning (*see glossary*) to ethylene glycol. There was no restriction on the context of the poisoning or heterogeneity of the participants (e.g., special populations such as pediatric, chronic kidney disease, pregnancy).

-Types of interventions: all types of ECTR if instituted at least partially for the purpose of poison removal. Studies in which ECTR was instituted exclusively for other indications such as kidney replacement therapy for acute kidney injury or albumin dialysis for hepatic failure will be excluded.

-Types of comparators: standard care (defined above) without ECTR (pre-defined, see Table above)

-Types of outcomes: patient-important outcomes ranked critical or important for decision-making were: inpatient mortality (critical), dialysis dependence at 3 months (critical), irreversible neurological damage (critical), AKI requiring short-term dialysis (critical), length of ICU stay (important), length of hospital stay (important), cost (important), serious complication of catheter insertion (critical), serious complications of ECTR (critical), complications related to ethanol as antidote. Dialyzability was reported as a separate outcome.

Search strategy: a search strategy was first developed in Pubmed/MEDLINE and then translated into the other databases, as appropriate. The following electronic databases were searched: Medline/PubMed, EMBASE, Cochrane Library (Review and Central), Conference proceedings/meeting abstracts of the EAPCCT and NACCT annual meetings (since 2002). All databases were searched from the date of inception to March 1st 2019. Bibliographies of all full-text articles were manually searched for additional papers. There were no language exclusion criteria nor any other publication restrictions.

Search strategy in Pubmed/MEDLINE:

1. ((kidney or renal) replacement therapy).tw
2. dialysis.tw
3. h?moperfusion.tw
4. h?mofiltration*.tw
5. h?modialysis.tw
6. h?modialfiltration.tw
7. h?moadsorption.tw
8. plasmaph?resis.tw
9. mars.tw
10. prometheus.tw
11. (liver support device*).tw
12. C?RT.tw
13. C?VH*.tw
14. (exchange transfusion).tw
15. (plasma exchange).tw
16. or/1–15
17. ethylene glycol.tw
18. 16 and 17

Literature search: Standard care
As mentioned above, if the initial search of the literature did not identify comparative studies in which ECTR was measured against standard care alone (with or without an antidote, if applicable), a specific search for the control group was developed.[6]

Eligibility criteria for study selection were based on:

-Types of study design: one-arm studies, cohorts, case series, case reports (if the former designs were not identified)

-Types of participants: all patients with severe poisoning to ethylene glycol (pre-defined). There was no restriction on the context of the poisoning (acute, acute-on-chronic or chronic) or heterogeneity of the participants (e.g., special populations such as pediatric, chronic kidney disease, hepatic insufficiency, pregnancy).

-Types of outcomes: patient-important outcomes (as defined above)

Search strategy: a search strategy was first developed in Pubmed/MEDLINE and then translated into the other databases, as appropriate. The following electronic databases were searched: Medline/PubMed, EMBASE, Cochrane Library (Review and Central), Conference proceedings/meeting abstracts of the EAPCCT and NACCT annual meetings (since 2002). All databases were searched from the date of inception to March 19th, 2019. Bibliographies of all full-text articles were manually searched for additional papers. There were no language exclusion criteria nor any other publication restrictions.

Search strategy in Pubmed/MEDLINE:

1. overdos*.tw
2. poisoning*.tw
3. toxicit*.tw
4. intoxicat*.tw
5. or/1–4
6.ethylene glycol.tw
7. 5 and 6

Literature search: ECTR harms
Potential complications of ECTRs, either from catheterization or from the procedure itself, were comprehensively searched in PubMed/Medline for the best available evidence (randomized controlled trials and large cohort studies). Non-poisoning data were accepted, although adapted to acute and short-term scenarios (e.g., long-term infectious risks of catheters were not considered). The keyword search strategy was: ((kidney or renal) replacement therapy) or dialysis or h?moperfusion or h?mofiltration* or h?modialysis or h?modialfiltration or h?moadsorption or plasmaph?resis or (plasma exchange) or mars or prometheus or (liver support device*) or C?RT or C?VH* or (exchange transfusion)) AND (complication OR safety OR side effect OR adverse event OR harm OR adverse effect)

Update of literature searches
This search strategy was reconducted on February 19th, 2022, and pertinent articles not initially identified were submitted to the workgroup to assess if they modified the recommendations. Some research that was published in abstract form on initial selection were later identified in the full text version and later updated (e.g., [7, 8]).

Screening of articles

All citations were entered into Endnote (version X9, Clarivate Analytics Inc., Philadelphia, PA). Three experienced workgroup members (D.M.R, D.M.W., and M.G.) screened the study titles and abstracts independently in duplicate to determine eligibility for full-text assessment. Any citation that was considered of interest was included for the full-text assessment. Prior to the full-text screening, all studies published in non-English languages were translated in English by designated translators. Subsequently, the same members examined the full texts to assess inclusion and exclusion criteria. Disagreements were resolved by consensus or by involvement of the methodologist (V.L.).

Data management and extraction

Relevant data were extracted from Endnote and abstracted into a standardized data extraction tool using Microsoft Excel software (Version 2102). One chair (M.G.) was responsible for the master copy. Four authors (D.M.R., S.G., M.R. and M.G.) extracted the data into Microsoft Excel software (version 2102); The methodologist (V.L.) reviewed all versions, resolved inconsistencies, and consolidated the data in one master flowsheet.

The following data for each eligible study were extracted into the standardized data extraction form:

-Study characteristics: first author, publication year, study design, number of treatment arms

-Baseline patient characteristics: age, gender, population size, kidney function, comorbidities

-Exposure: route of exposure, dose, length of exposure, intent, time from exposure to admission, coingestion of ethanol

-Clinical characteristics of poisoning: Hypotension, mental status alteration, coma, cerebral edema, seizure, acid-base abnormalities, oxalate crystals in the urine, osmol gap, respiratory depression, AKI, serum creatinine, and blood sampling of ethylene glycol and metabolites (on admission, peak value)

-Non-ECTR management: antidote (ethanol and/or fomepizole), vitamins, vasopressors, mechanical ventilation, decontamination

-ECTR: type, indication, interval between admission and ECTR, number of sessions, duration of session, catheter, prescription (e.g., anticoagulation, dialyzer, dialysate)

-Toxicokinetics (protein binding, volume of distribution, endogenous clearance, half-life (pre, per, and post-ECTR), extraction ratio, ECTR clearance, removal by ECTR, removal in urine, presence and magnitude of rebound. These were calculated by the data extractors if not reported.

-Clinical outcome: death, length of hospital stay, reported improvement during ECTR, length of ICU stay, neurological sequelae, length of mechanical ventilation, kidney outcomes.

Dialyzability

Dialyzability was defined a priori as the ability of any ECTR to remove a clinically significant percentage of the total body burden of the poison.[3] Different criteria were used to semi-quantitively categorize the dialyzability of the poison for each ECTR (Table S4).

Supplemental Table S4. EXTRIP criteria for assessing dialyzability

**Dialyzability†**	**Primary criterion**	**Alternative criterion 1**	**Alternative criterion 2**	**Alternative criterion 3**
	**% Removed‡**	**CL_{ECTR} / CL_{TOT} (%)§**	**$T_{1/2\ ECTR}$ / $T_{1/2}$ (%)¶**	**RE_{ECTR} / RE_{TOT} (%)#**
**D**, Dialyzable	>30%	>75%	<25%	>75%
**M**, Moderately dialyzable	>10-30%	>50-75%	>25-50%	>50-75%
**S**, Slightly dialyzable	≥3-10%	≥25-50%	≥50-75%	≥25-50%
**N**, Not dialyzable	<3%	<25%	<75%	<25%

† Applicable to all modalities of ECTR, including hemodialysis, hemoperfusion, hemofiltration.
‡ Corresponds to % removal of ingested dose (adjusted for bioavailability) or total body burden, adjusted for a 6-hour ECTR period.
§ Corresponds to the clearance by ECTR (CL_{ECTR}) compared to the total clearance (CL_{TOT} i.e., endogenous + ECTR clearance)
¶ Corresponds to the apparent half-life during ECTR ($T_{1/2\ ECTR}$) compared to the apparent half-life off ECTR ($T_{1/2}$).
# Corresponds to the amount removed by ECTR (RE_{ECTR}) compared to the amount eliminated from the body (RE_{TOT}) measured during the same period.
These criteria should only be applied if measured or calculated (not reported) endogenous $T_{1/2}$ is > 4h (otherwise, the benefit of ECTR is not likely to be considered clinically relevant). Furthermore, the primary criterion is preferred for poisons having a large V_D (>1-2L/Kg)

Quality assessment of individual studies

The quality of reporting and risk for bias of each included study was assessed independently by two workgroup members (D.R. and M.G). Disagreements were resolved by consensus or involvement of the methodologist (V.L.). Risk of bias was assessed using the Cochrane Risk of Bias Tool for RCTs and the Risk of Bias Instrument for Non-randomized Studies – of Interventions (ROBINS-I).[9, 10]

The quality of individual studies reporting on toxicokinetic outcomes was assessed according to a pre-defined set of criteria (Table S5) and then summarized into a quality of the overall evidence (Table S6). If the latter was judged low or very low, literature from non-poisoning contexts was also considered, such as CKD pharmacokinetics, animal, and *in vitro* studies.

Supplemental Table S5: Quality of individual studies for toxicokinetic outcomes.

**Quality of individual studies**	**Interpretation and application to individual studies**
High	Sufficient TK/PK data present; % removed is reported or can be calculated; reported calculations are appropriate.
Moderate	Sufficient TK/PK data present, but % removed is NOT reported or CANNOT be calculated; reported calculations (e.g., CL_{EC}/CL_{TOT}) are appropriate.
Low	Sufficient PK parameters may be reported, but supporting data absent or suspect, reported calculations inappropriate, or other serious limitations exist.
Very Low	Sufficient PK parameters and supporting data not adequately reported, questionable or no calculations reported. However, based on theoretical knowledge of V_D, protein binding, CL_{SYS}, molecular weight, etc., some assumptions can be made about dialyzability.
Reject	Questionable parameters reported with no supporting data, fatal flaw in study design.

Supplemental Table S6. Quality of evidence for toxicokinetic outcomes

**Quality of evidence**	**Reporting**	**Interpretation**
High	A	We are confident that the true effect lies close to our estimate of the effect.
Moderate	B	The true effect is likely to be close to our estimate of the effect, but there is a possibility that it is substantially different.
Low*	C	The true effect may be substantially different from our estimate of the effect.
Very Low*	D	Our estimate of the effect is just a guess, and it is very likely that the true effect is substantially different from our estimate of the effect*.

**If the quality of the evidence is low or very low, literature from non-poisoning contexts may be used, such as pharmacokinetic studies in ESKD populations, animal, and in-vitro studies.*

Data synthesis

For dichotomous outcome measures, pooled estimate of the proportion of patients across studies with the corresponding 95% confidence interval (CI) was calculated. For continuous outcomes, a pooled estimate of the mean and a corresponding 95% CI or the median with first and third quartiles, was calculated, as appropriate. Due to the presumed lack of comparative studies on the effect of ECTR vs standard care alone, no meta-analysis was planned *a priori*.

Summary of evidence and quality of evidence

Evidence summaries for each question were prepared by the workgroup members in collaboration with the methodologist. For a clinical question to be formally developed into a recommendation, the workgroup agreed *a priori* that a minimum of 3 reported cases describing clinical outcomes for a specific poison was required.

In the absence of direct comparison between the intervention (standard care plus ECTR) and comparator (standard care alone), the members selected the publications reporting controls that most closely resembled patients from the ECTR group (especially with regards to severity of poisoning). For example, controls admitted to the ICU were considered more likely to be comparable to the ECTR cohort than patients reported to poison control centers.

The quality of the evidence (Figure S1) was initially assessed for each critical and important outcome, and then for each recommendation using the Grading of Recommendations Assessment, Development and Evaluation (GRADE) approach.[11, 12] The quality of evidence was assessed across the domains of risk of bias, consistency, directness, precision and publication bias. Additional domains were considered when appropriate. Quality was adjudicated as high (further research is very unlikely to change our confidence in the estimate of effect), moderate (further research is likely to have an important impact on our confidence in the estimate of effect and may change the estimate), low (further research is very likely to have an important impact on our confidence in the estimate of effect and is likely to change the estimate), or very low (very uncertain about the estimate of effect). GRADE evidence profile tables were developed in GRADEpro Guideline Development Tool (GRADEpro GDT: GRADEpro Guideline Development Tool [Software]. McMaster University, 2015 (developed by Evidence Prime, Inc.). Available from gradepro.org). The summaries of evidence were reviewed by all workgroup members prior to drafting recommendations.

Supplemental Figure S1: Approach to and implications of rating the quality of the evidence and strength of recommendations using the GRADE methodology.

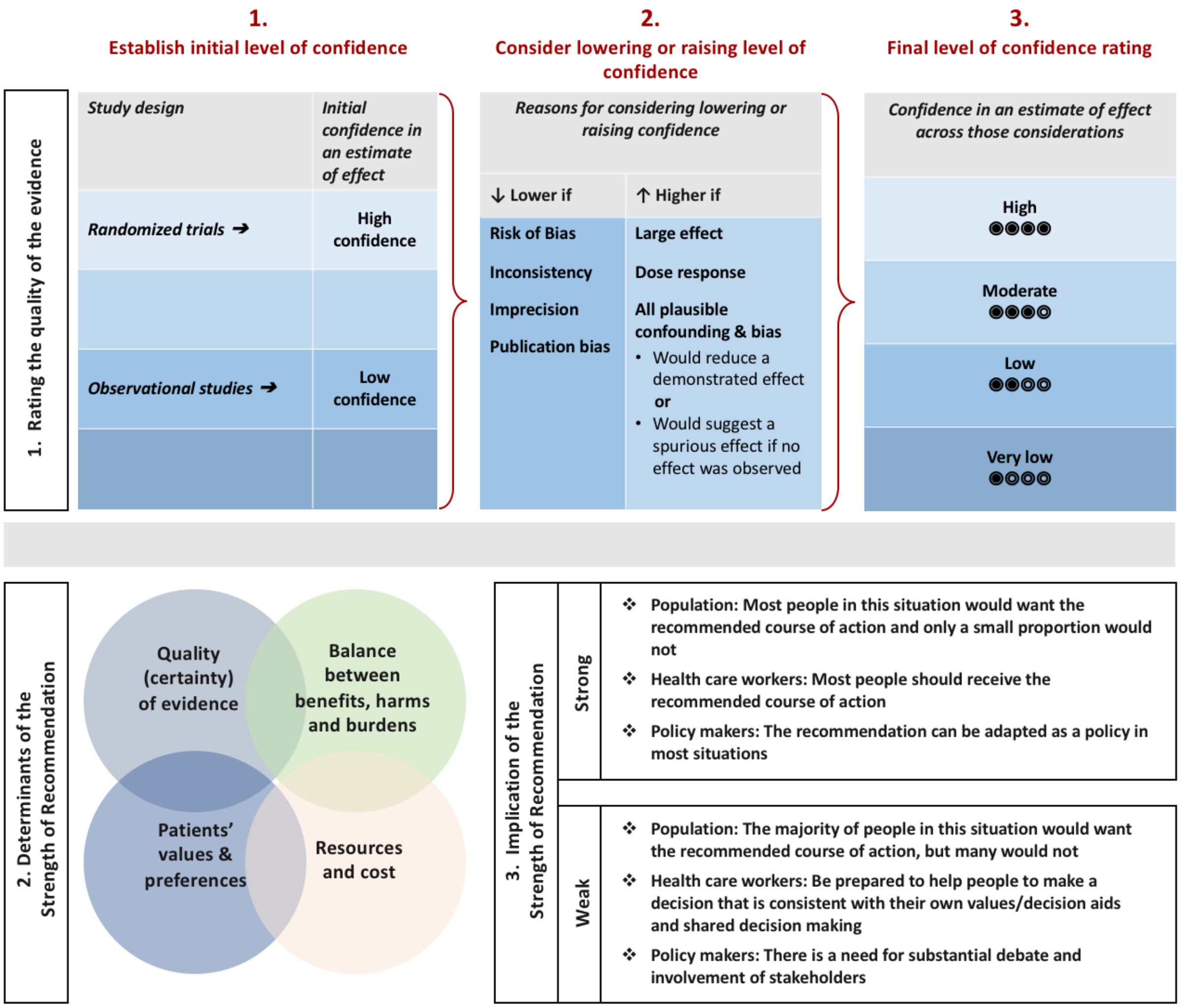

Unrestricted use of the figure granted by the GRADE working group.

Development of clinical recommendations

The workgroup considered the core elements of the GRADE approach for the decision process, including the balance between desirable and undesirable effects and the quality of evidence. Additional domains were acknowledged where applicable (feasibility, resource use, acceptability). A web-based international survey performed by the EXTRIP workgroup provided the main source of evidence to address other important considerations such as resource use, costs, and availability of ECTR worldwide [4]. This survey also informed potential implementation issues. EXTRIP discussed organizational barriers in health care centers that may not have the resources and technical expertise to adopt the recommendations. Recommendations will also address situations where ECTR was previously performed but no longer supported.

For all recommendations, the workgroup members voted to reach agreement for final recommendations. The voting process followed the same set of rules as for EXTRIP-1 (i.e., anonymous online voting consisting of two-round modified Delphi with each statement voted on a 9-point Likert scale and final results interpreted according to EXTRIP voting rules based on median, lower or upper quartile (LQ or UQ) as appropriate, and disagreement index (DI) as calculated using RAND/UCLA Appropriateness Method (Figure S2)) and was performed using SimpleSurvey software.

Supplemental Figure S2: Voting process for recommendations.

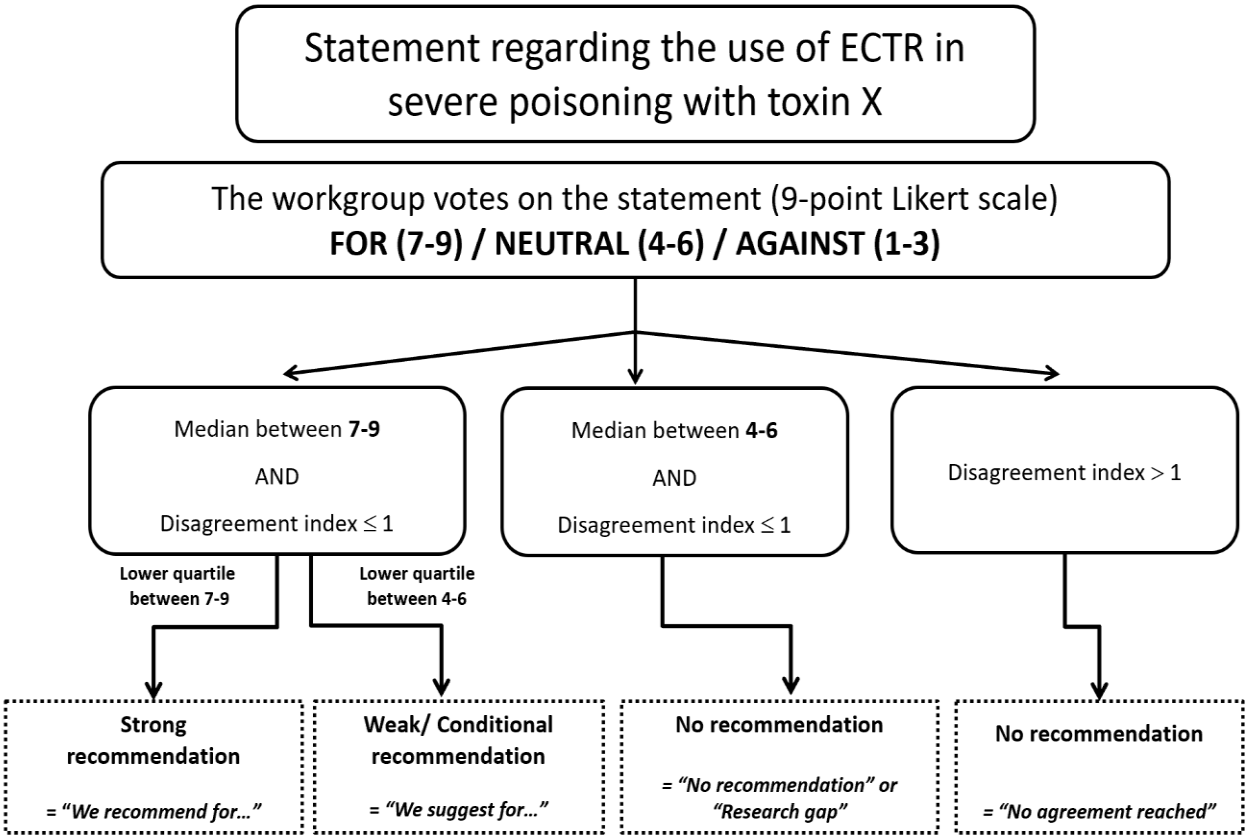

These recommendations can be formulated for or against a course of action (the figure only shows voting results FOR a course of action)

All recommendations were labelled as either "strong" or "weak/conditional" according to the GRADE approach. The words "we recommend" indicate strong recommendations and "we suggest" indicate weak/conditional recommendations. Figure S1 provides the suggested interpretation of strong and weak/conditional recommendations for patients, clinicians, and health care policy makers. In summary, a "strong" recommendation implies that most individuals in this situation would want the recommended course of action and only a small proportion would not, while a "weak/conditional" recommendation means that the majority of individuals in this situation would want the suggested course of action, but many would not. The latter recognizes that different choices will be appropriate for different patients and that clinicians must help each patient to arrive at a management decision consistent with their own values and preferences.

High-quality evidence was expected to be lacking for the majority of recommendations. According to GRADE guidance, strong recommendations in the setting of lower-quality evidence were only assigned when the workgroup members believed they conformed to one or several paradigmatic conditions. As per GRADE guidance on discordant recommendations[13], two paradigmatic situations presented in the development of EXTRIP-2 guideline: 1) low-quality evidence suggested benefit in a life-threatening situation (with evidence regarding harms being low or high), and 2) when low-quality evidence suggested benefit and high-quality evidence suggested harm.

If the workgroup could not make a recommendation for or against a particular management strategy due to either 1) a close balance between the benefits and harms (no recommendation), or 2) insufficient evidence making a recommendation too speculative (research gap), either a "no recommendation" or "research gap" recommendation was formulated (a "neutral recommendation" interpreted as a "reasonable course of action" is no longer acceptable). Although there is arguably ongoing need for research on virtually all of the topics

considered in this guideline, “Research Needs” were noted for recommendations in which the need was believed by the workgroup to be particularly relevant.

The entire workgroup gathered in Montreal, Canada in November 2019 for the presentation of evidence summaries and the development of the recommendations for the ten new poisons. The subgroup assigned to a specific poison participated in the preparation of the draft guideline in collaboration with co-chairs.

SUPPLEMENTAL TABLES AND FIGURES FROM THE MANUSCRIPT

Supplemental Table S7: Reports of toxicity in patients ingesting < 100mL equivalent of pure ethylene glycol

**Reference**	**Age and sex**	**Dose (mL)**	**Dose (mL/kg)**	**Delay to presentation (h)**	**ETOH Co-ingestion**	**EG concentration (mmol/L)**	**Antidote**	**Ingestion to ECTR (h)**	**ECTR**	**Outcome**
Mégarbane et al., 2001 [14]	27 F	50	?	12	No	10	ETOH	?	No	On arrival, HCO_3^- = 6 mmol/L, pH=7.12
Spillane et al., 1991 [15]	22 M	75	?	168	No	?	No	168	HD	AKI = 28 days
Tanasecu et al., 2014 [16]	32 M	30	?	14	No	?	ETOH	?	HD	AKI with creatinine = 603 µmol/L
Sabeel et al., 1995 [17]	?	250	?	2	?	4.7	?	?	?	AKI = 28 days
	67 ?	30	0.5	23.5	No	0.1	ETOH	29.5	HD	AKI = 91 days
	24 ?	100	1.4	3.0	?	3.3	ETOH	23.5	HD	AKI = 21 days
	42 ?	100	1.1	3.5	?	2.2	ETOH	23.0	HD	AKI = 35 days
Kidawa et al., 1987 [18]	Between 20-61	75	?	?	?	0	?	140	HD	AKI = 13 days
		75	?	?	?	0.5	?	54	HD	Died
		75	?	?	?	1.5	?	24	HD	AKI =22 days
Wideman et al., 1946 [19]	42 M	<60	?	72	?	?	?	?	?	Death at 6 days
Rydel et al., 2002 [20]	50 F	60	1.0	68	?	0	?	?	?	Developed acidosis & AKI; creatinine >194 µmol/L
Krivoshapkin et al., 1983 [21]	? n=25	25-120	?	0 to 48	?	?	All ETOH	0 to 48	All HD	6/25 died, 1 patient died with ≤ 50mL pure EG

Legend: ETOH: Ethanol, N/A: Not available, AKI, Acute kidney injury, EG: Ethylene glycol, ECTR: Extracorporeal treatment, HCO_3^-: bicarbonate concentration, HD: hemodialysis

Supplemental Table S8: Studies comparing the effect of different modalities of ECTR on clinical outcomes

Study	Year, country, type of study, type of population	Comparison	Baseline clinical comparison and outcomes	Conclusion	Interpretation
Swiderska et al., 2013 [22]	2010, Poland<br>(n=98) received ECTR<br><br>Global severity of included population: Death (n=26/98)	HD (n=83) vs CKRT (n=15)	Not compared at baseline<br><br>Patients treated HD 20/83 = 24% mortality while those treated with CKRT had 6/15= 40% mortality	Similar mortality when comparing the 2 groups	The groups were not compared at baseline; thus, no conclusion can be formulated<br><br>Did the indication of the type of ECTR vary between groups (eg hypotensive patients receiving CKRT rather than HD)?
Rydel et al., 2002 [20]	1994-1998, Chicago (2 community hospitals), retrospective<br>(N=10) received ECTR<br><br>Global severity of included population: Death (n=2/10)	CHD (n=5) vs IHD (n=5)	Data presented according to time to presentation and no details are provided to compare CHD and IHD except:<br>-Undetectable plasma ethanol concentration in all patients<br>-Patients who received CHD presented usually < 12h after ingestion and those who received IHD presented usually > 12h after ingestion<br>-CHD was stopped when osmol gap =0. IHD stopped when EG concentration = 8 mmol/L<br><br>Mortality was 2/5 in IHD and 0/5 in CHD. Sequalae occurred in 3/5 in IHD group and 0/5 in CHD = composite outcome (P-value 0.008)<br><br>Time to recovery of AKI was 5 days in CHD vs 11.8 days in IHD	Higher sequelae or mortality in the IHD group as compared to the CHD group<br><br>Time to recovery of AKI was longer in IHD group.	Confounded by the time to ECTR. Probable confounding-by-indication?<br><br>Did the administered antidote vary between groups?<br><br>What is the impact of different cut-offs between the 2 groups to stop ECTR?<br><br>Underpowered
Sabeel et al., 1995 [17]	Lund University Hospital, Sweden; 1982-1991<br><br>Retrospective chart review	HD+PD (n=7) vs HD (n=11)<br><br>Global severity of included population: Death was 1/18 (5.6%), and long-term CKD not requiring dialysis was 1/18 (5.6%)	Comparable mortality between groups (1/7 (14.3%) in the HD-PD group vs 0/11 (0%) in the HD group).<br><br>AKI was similar between groups: 6/7 (85.7%) in the HD-PD group vs 8/11 (72.7%) in the HD group, of which 5/6 and 6/8 required dialysis, respectively.<br><br>EG was removed more rapidly, and acidosis corrected more rapidly in the HD-PD group.<br><br>The HD-PD group had higher creatinine on discharge as compared to the HD group (252 µmol/L vs 149 µmol/L, respectively) and had a longer time to normalization of kidney function (23 vs 7.9 weeks, respectively) (P-value 0.015).<br><br>Long term CKD occurred in 1 patient in the ECTR group.	Baseline characteristics reported were comparable.<br><br>Indication of PD in addition to HD was for cases with more advanced toxicity symptoms and severity of acidosis. Indeed, the HD-PD group were more acidotic (BE -27 vs -16mmol/L in HD group), had a higher EG concentration (18.5 vs 10mmol/L in HD group), and a higher creatinine 72h after admission (521 vs 413 respectively, despite having a lower creatinine on admission (144 vs 170µmol/L in HD group)).<br><br>Clinically, the HD-PD group tended to be sicker (3/7 vs 1/11 had convulsions (NS), 6/7 vs 5/11 required mechanical ventilation (NS)).	Confounding-by-indication<br><br>Underpowered<br><br>P-value not calculated for continuous variables

Legend: HD: Hemodialysis, CKRT: Continuous kidney replacement therapy, CHD: Continuous hemodialysis, HD: Hemodialysis, IHD: Intermittent hemodialysis, PD: Peritoneal dialysis, CKD: Chronic kidney disease, AKI: Acute kidney injury, EG: Ethylene glycol, ECTR: Extracorporeal treatment

Supplemental Table S9: impact of time to ECTR initiation on clinical outcomes

Study	Year, country, type of study, type of population	Comparison	Baseline comparison	Conclusion	Interpretation
Lung et al., 2015 [23]	1999-2008, CA, U.S.<br><br>Retrospective case review (non-consecutive)<br><br>(n=121) patients identified through lab database with documented plasma EG concentration, pH, creatinine, time to treatment and follow-up<br><br>Global severity of included population: Death (n=9) or AKI requiring HD for more than 3 days (n=50) = 59/121	HD (n=102)<br><br>Time to ECTR: Earlier vs later time to dialysis (< 6h vs ≥ 6h)	Not detailed	HD administered ≥ 6h was associated with lower odds of AKI (OR 0.36 with 95% 0.15-0.87) adjusted for age and gender but not pH since it was too strongly associated with AKI (strong predictor)	Confounding likely: time to dialysis likely associated with the severity of the disease<br><br>Model cannot be fully adjusted (i.e., not adjusted for pH which is a strong predictor of worse outcome) thus results are further confounded by known factors.
Krivoshapkin et al., 1983 [21]	1977 to 1981, Russia<br><br>(n=34)	Dialysis (n=34)<br><br>Time to ECTR	Time to ECTR:<br><12h = 0/11 deaths, 12-24h = 2/15 deaths. >24h = 6/8 deaths<br><br>Fisher exact test: p=0.0004<br><br>Subgroup analysis shows that the group "more than 24h" is statistically different than the two others separately or combined<br>< 12h vs > 24h: p=0.001<br>12-24h vs > 24h: p=0.006<br>OR<br>< 24h vs > 24h: p=0.0005<br><br>There is **no statistical difference** between < 12h and 12-24h	Mortality is higher in the group receiving HD more than 24h after poisoning	Confounding likely: time to dialysis likely associated with time to other cointerventions

Legend: HD: Hemodialysis, IHD: Intermittent hemodialysis, AKI: Acute kidney injury, EG: Ethylene glycol, ECTR = Extracorporeal treatment

Supplemental Table S10: Fatalities in patients with a glycolate < 12mmol/L or anion gap (calculated as Na^{+}+K^{+}-Cl^{-}-HCO_3^{-}) below 28mmol/L

Reference	Age (y)	Sex	EG volume ingested (mL)	Ethanol co-ingestion	Time to admission (h)	HCO_3^- (mmol/L)	AG (mmol/L)	EG concentration (mmol/L)	Antidote	ECTR	Time from ingestion to ECTR (h)	Relevant outcome and notes
Tanasescu et al., 2014 [16]	50	M	500	?	8	13.2	11.6*	?	Ethanol	HD	?	Died. No data on what happened
Munro et al., 1967 [32]	75	M	300	?	16	19	26.4	?	None	HD	23.5	Died 47h after ingesting EG
Tanasescu et al., 2014 [16]	77	F	?	?	?	13.4	23.3*	?	Ethanol	HD	?	Died. No data on what happened

Legend: EG: Ethylene glycol, OG: Osmol gap, AG: Anion gap, ECTR: Extracorporeal treatment, HCO_3^-: bicarbonate concentration, HD: Hemodialysis.

* It was not reported how the calculation of the anion gap was done but was assumed to have been without using K.

Supplemental Table S11: Correlation between the osmol gap and the ethylene glycol concentration in mmol/L

Reference	Sample data points	Equation	R^2
Hovda et al., 2004 [24]	20	OG = 0.84xEG + 17.8	0.88
Hovda et al., 2011 [25]	59	OG = 1.05xEG + 21	0.76
Greene et al., 2020 [26]	133	OG = 1.18xEG - 2.5	0.94
Jacobsen et al., 1982 [27]	5	OG= 0.77xEG + 16.4	0.98
EXTRIP systematic review	107	OG = 0.96xEG + 21	0.78
Lund et al., 1983 [28]	5	Osmolality = 0.97xEG + 287	0.94
Porter et al., 2001 [29]	16	OG = 0.89xEG + 15.4	0.93
Khajuria et al., 2005 [30]	34	OG = 1.00xEG + ?	?
Montjoy et al., 2010 [31]	9	OG = 1.22xEG + 26.7	0.53

Legend: EG: Ethylene glycol concentration, OG: Osmol gap

Supplemental Table S12: Antidote dosing

Antidote		Loading dose	Maintenance dose			
			**Nondrinker**	**Chronic drinker**	**HD, nondrinker**	**HD, drinker**
**Ethanol**	43% oral solution	1.8 mL/kg	0.2 mL/kg/h	0.5 mL/kg/h	0.5 mL/kg/h	0.8 mL/kg/h
	10% IV solution	7.5 mL/kg	0.8 mL/kg/h	2.0 mL/kg/h	2.1 mL/kg/h	3.3 mL/kg/h
	Dialysate	300 mL 95% ethanol added to 4.5L of acid concentrate, ultrafiltrate flow = 500mL/min				
**Fomepizole**		15 mg/kg	10mg/kg q 12h x 4 doses, then 10-15mg/kg q 12h		10mg/kg q 4h (q8h in CKRT) or 1mg/kg/h	

Legend: HD: Hemodialysis, CKRT: Continuous kidney replacement therapy, IV: Intravenous

Reproduced with modifications from [33, 34, 35, 36]

*Supplemental Figure S3: Modeling scenario of time to safe ethylene glycol concentration**

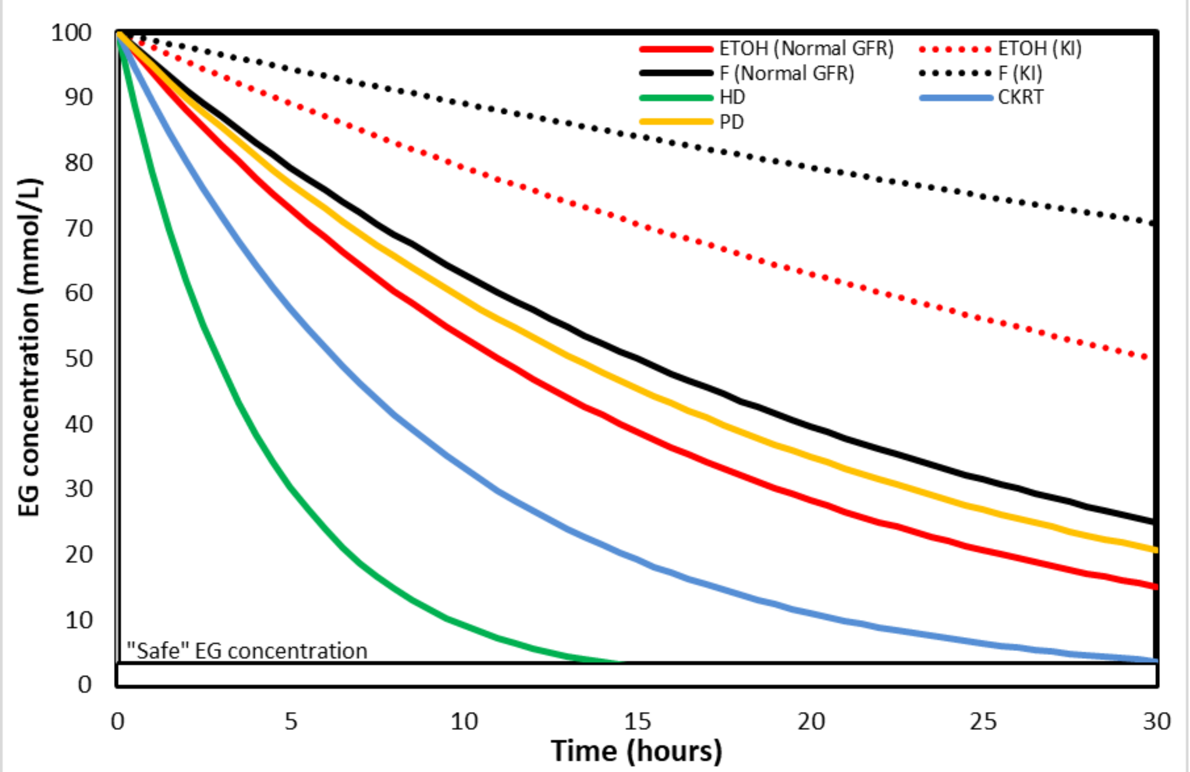

Condition	Assumed $T_{1/2}$ (hours)	Time to reach <3.2 mmol/L (hours)
**Ethanol (Normal GFR)**	11	55
**Ethanol (Kidney impairment)**	30	149
**Fomepizole (Normal GFR)**	15	75
**Fomepizole (Kidney impairment)**	60	297
**Hemodialysis**	2.9	15
**Peritoneal dialysis**	13.2	66
**Continuous kidney replacement therapy**	8.5	32

*Assuming first order decay, initial [EG]= 100 mmol/L, safe [EG] = 3.2 mmol/L, and half-lives taken from Table 1 and Table 2 in main manuscript

Legend: KI: kidney impairment (including acute kidney injury and chronic kidney disease); F: Fomepizole; CKRT: Continuous kidney replacement therapy; PD: Peritoneal dialysis; HD: Hemodialysis; GFR: Glomerular filtration rate; ETOH: ethanol; EG: ethylene glycol.

GLOSSARY

-*Acute liver failure*: (a) the presence of hepatic encephalopathy of any degree; (b) evidence of moderately severe coagulopathy [i.e., international normalized ratio (INR) ≥1.5]; (c) presumed onset of acute illness of <26 weeks; and (d) the absence of cirrhosis
-*Acute liver injury*: (a) evidence of moderately severe coagulopathy (INR ≥2.0); (b) presumed onset of acute illness <26 weeks; and (c) the absence of cirrhosis
-*Altered mental status*: poison-induced impairment in at least one brain function (cognition, alertness, or orientation), in the absence of another cause
-*Clearance*: The volume of blood (or solvent) cleared of poison per unit time, typically reported in units of mL/min. Importantly, CL_{ECTR} represents solute clearance due exclusively to ECTR and is independent of endogenous clearance (CL_{ENDO}; the sum of underlying renal and non-renal clearances). CL_{TOT} refers to total clearance and is the sum of CL_{ECTR} and CL_{ENDO}
-*Coma*: a deep state of unconsciousness as defined per the American Academy of Neurology
-*Dialyzability*: This term reflects the ability of ECTR to remove a clinically significant percentage of the total body burden of the poison
-*Extracorporeal treatment (ECTR)*: A treatment, occurring outside the body, which promotes poison removal by mechanisms different from endogenous pathways. ECTR includes HD, continuous renal replacement therapy, extended dialysis, peritoneal dialysis (although technically occurring in the body), hemofiltration, hemodiafiltration, hemoperfusion, therapeutic plasma exchange and albumin/ “liver” dialysis
-*Kidney impairment*: CKD as stage 3B, 4, or 5 CKD (i.e., eGFR < 45 mL/min/1.73m^2) or AKI as KDIGO stage 2 or 3 AKI. In the absence of a baseline creatinine, a GFR < 45 mL/min in adults; in children with no baseline creatinine, the use of KDIGO criteria of AKI stage 2 and 3 after imputing a baseline serum creatinine using the Schwartz 2009 formula assuming 120 mL/min of "normal" eGFR. The presence of oligo/anuria unresponsive to fluid resuscitation should be considered as impaired kidney function, regardless of serum creatinine concentration
-*Poison*: A xenobiotic (exogenous chemical, including medications and drugs) or an endogenously found chemical (e.g., iron, copper, vitamins) resulting from exogenous exposure with the potential to cause toxicity.
-*Poisoning*: Exposure to a poison causing or capable of causing toxicity, regardless of intent. It includes intoxication, toxicity, and overdose
-*Refractory bradycardia*: per age-related defined standards, after vasopressor/inotropic support
-*Refractory hypotension*: per age-related defined standards, after adequate fluid challenge and vasopressor/inotropic support
-*Serious complications of catheter insertion*: these include hemothorax, pneumothorax, hemomediastinum, hydromediastinum, hydrothorax, subcutaneous emphysema retroperitoneal hemorrhage, embolism, nerve injury, arteriovenous fistula, tamponade, and death if occurring within 72 hours of insertion. Hematoma and arterial puncture were judged not serious and thus excluded from this composite outcome. DVT and infectious complications were not included considering the short duration of catheter use.
-*Serious complications of ECTR*: for hemodialysis and CKRT, these include air emboli and shock; minor bleeding from heparin, transient hypotension, and electrolytes imbalance were judged not serious. For hemoperfusion: these include severe thrombocytopenia, major bleeding, and hemolysis; transient hypotension, hypoglycemia, hypocalcemia, and thrombocytopenia were judged not serious. For therapeutic plasma exchange, these include citrate toxicity, severe allergic reaction, dysrhythmia, and vasovagal reaction; hypotension, hypocalcemia, and urticaria were judged as not serious. They were attributed to the ECTR if occurring within 72 hours of initiation.
-*Severe poisoning*: Exposure to a poison causing or capable of causing, if left untreated, end-organ damage.
-*Shock or end-organ compromise*: Hypotension (systolic blood pressure < 90 mmHg or mean blood pressure < 65 mmHg) with the presence of cellular ischemia as evidenced by increased lactate concentration, acute kidney injury (AKI) as defined by the Kidney Disease Improving Global Outcomes (KDIGO) guideline, increased troponin, altered mental status, or decreased capillary refill

REFERENCES

1. Institute of Medicine Committee on Standards for Developing Trustworthy Clinical Practice G. In: Graham R, Mancher M, Miller Wolman D, Greenfield S, Steinberg E, editors. Clinical Practice Guidelines We Can Trust. Washington (DC): National Academies Press (US); 2011.
2. Ghannoum M, Nolin TD, Lavergne V, Hoffman RS. Blood purification in toxicology: nephrology's ugly duckling. Adv Chronic Kidney Dis. 2011;18:160-6. Epub 2011/05/03.
3. Lavergne V, Nolin TD, Hoffman RS, Roberts D, Gosselin S, Goldfarb DS, Kielstein JT, Mactier R, MacLaren R, Mowry JB, Bunchman TE, Juurlink D, Megarbane B, Anseeuw K, Winchester JF, Dargan PI, Liu KD, Hoegberg LC, Li Y, Calello D, Burdmann EA, Yates C, Laliberte M, Decker BS, Mello-Da-Silva CA, Lavonas E, Ghannoum M. The EXTRIP (Extracorporeal Treatments In Poisoning) workgroup: Guideline methodology. Clin Toxicol. 2012;50:403-13.
4. Bouchard J, Lavergne V, Roberts DM, Cormier M, Morissette G, Ghannoum M. Availability and cost of extracorporeal treatments for poisonings and other emergency indications: a worldwide survey. Nephrol Dial Transplant. 2017;32:699-706.
5. Moher D, Shamseer L, Clarke M, Ghersi D, Liberati A, Petticrew M, Shekelle P, Stewart LA, Group P-P. Preferred reporting items for systematic review and meta-analysis protocols (PRISMA-P) 2015 statement. Syst Rev. 2015;4:1. Epub 2015/01/03.
6. Beaulieu J, Roberts DM, Gosselin S, Hoffman RS, Lavergne V, Hovda KE, Megarbane B, Lung D, Thanacoody HKR, Ghannoum M. Treating ethylene glycol poisoning with alcohol dehydrogenase inhibition, but without extracorporeal treatments: a systematic review. Clinical Toxicology. 2022.
7. Grigorasi G, Nistor I, Corlade-Andrei M, Siriopol D, Cimpoesu CD, Covic A. Analysis of prognostic factors for survival in a large cohort of ethylene glycol intoxication cases. Nephrology Dialysis Transplantation. 2018;33 (Supplement 1):i50-i1.
8. Grigorasi GR, Nistor I, Corlade-Andrei M, Voroneanu L, Siriopol D, Apetrei M, Cimpoesu DC, Covic A. Outcomes of death and prolonged renal insufficiency in ethylene glycol poisoned patients. International urology and nephrology. 2022;54:149-55. Epub 2021/03/20.
9. Higgins JPT, Thomas J, Chandler J. Cochrane Handbook for Systematic Reviews of Interventions. 2nd ed. Chichester (UK): John Wiley & Sons; 2019.
10. Sterne JA, Hernan MA, Reeves BC, Savovic J, Berkman ND, Viswanathan M, Henry D, Altman DG, Ansari MT, Boutron I, Carpenter JR, Chan AW, Churchill R, Deeks JJ, Hrobjartsson A, Kirkham J, Juni P, Loke YK, Pigott TD, Ramsay CR, Regidor D, Rothstein HR, Sandhu L, Santaguida PL, Schunemann HJ, Shea B, Shrier I, Tugwell P, Turner L, Valentine JC, Waddington H, Waters E, Wells GA, Whiting PF, Higgins JP. ROBINS-I: a tool for assessing risk of bias in non-randomised studies of interventions. BMJ. 2016;355:i4919. Epub 2016/10/14.
11. Guyatt GH, Oxman AD, Vist GE, Kunz R, Falck-Ytter Y, Alonso-Coello P, Schunemann HJ, Group GW. GRADE: an emerging consensus on rating quality of evidence and strength of recommendations. BMJ. 2008;336:924-6. Epub 2008/04/26.
12. Schunemann HJ, Oxman AG. Handbook for grading the quality of evidence and the strength of recommendations using the GRADE approach Hamilton, ONT2020 [cited 2020 March 13]. Available from: https://gdt.gradepro.org/app/handbook/handbook.html.
13. Andrews JC, Schunemann HJ, Oxman AD, Pottie K, Meerpohl JJ, Coello PA, Rind D, Montori VM, Brito JP, Norris S, Elbarbary M, Post P, Nasser M, Shukla V, Jaeschke R, Brozek J, Djulbegovic B, Guyatt G. GRADE guidelines: 15. Going from evidence to recommendation-determinants of a recommendation's direction and strength. J Clin Epidemiol. 2013;66:726-35. Epub 2013/04/11.
14. Mégarbane B, Baud F. Acute ethylene glycol intoxication: A case report. Medecine Therapeutique. 2001;7:163-7.
15. Spillane L, Roberts JR, Meyer AE. Multiple cranial nerve deficits after ethylene glycol poisoning. Ann Emerg Med. 1991;20:208-10. Epub 1991/02/01.
16. Tanasescu A, Macovei RA, Tudosie MS. Outcome of patients in acute poisoning with ethylene glycol--factors which may have influence on evolution. Journal of medicine and life. 2014;7 . 3:81-6.
17. Sabeel AI, Kurkus J, Lindholm T. Intensified dialysis treatment of ethylene glycol intoxication. Scandinavian journal of urology and nephrology. 1995;29:125-9. Epub 1995/06/01.
18. Kidawa Z, Trznadel K. Haemodialysis in the treatment of acute ethylene glycol poisoning. Biul WAM. 1987;30:1-13.

19. Widman C. A few Cases of Ethylene Glycol Intoxication. Acta Med Scand. 1946;126:295-306.
20. Rydel JJ, Carlson A, Sharma J, Leikin J. An approach to dialysis for ethylene glycol intoxication. Vet Hum Toxicol. 2002;44:36-9. Epub 2002/02/05.
21. Krivoshapkin VG, Shutov AM, Shcherbak EN, Lopatin AI, Krugliakov VV. Hemodialysis in antifreeze poisoning. Sov Med. 1983:89-91. Epub 1983/01/01.
22. Swiderska A, Sein Anand J. [Selected data of acute poisonings with ethylene glycol and methanol in Poland in the year 2010]. Przeglad Lekarski. 2013;70:479-84.
23. Lung DD, Kearney TE, Brasiel JA, Olson K. Predictors of Death and Prolonged Renal Insufficiency in Ethylene Glycol Poisoning. Journal of Intensive Care Medicine. 2015;30:270-7.
24. Hovda KE, Hunderi OH, Ovrebo S, Jacobsen D. Diagnosis in metabolic acidosis of unknown origin. Tidsskr Nor Laegeforen. 2004;124:3203-5. Epub 2004/12/21.
25. Hovda KE, Julsrud J, Ovrebo S, Brors O, Jacobsen D. Studies on ethylene glycol poisoning: one patient - 154 admissions. Clin Toxicol. 2011;49:478-84. Epub 2011/08/10.
26. Greene HR, Krasowski MD. Correlation of osmolal gap with measured concentrations of acetone, ethylene glycol, isopropanol, methanol, and propylene glycol in patients at an academic medical center. Toxicology Reports. 2020;7:81-8.
27. Jacobsen D, Bredesen JE, Eide I, Ostborg J. Anion and osmolal gaps in the diagnosis of methanol and ethylene glycol poisoning. Acta Med Scand. 1982;212:17-20. Epub 1982/01/01.
28. Lund ME, Banner W, Jr., Finley PR, Burnham L, Dye JA. Effect of alcohols and selected solvents on serum osmolality measurements. J Toxicol Clin Toxicol. 1983;20:115-32.
29. Porter WH, Rutter PW, Bush BA, Pappas AA, Dunnington JE. Ethylene glycol toxicity: the role of serum glycolic acid in hemodialysis. J Toxicol Clin Toxicol. 2001;39:607-15. Epub 2002/01/05.
30. Khajuria A, Krahn J. Osmolality revisited--deriving and validating the best formula for calculated osmolality. Clin Biochem. 2005;38:514-9.
31. Montjoy CA, Rahman A, Teba L. Ethylene glycol and methanol poisonings: case series and review. The West Virginia medical journal. 2010;106:17-23.
32. Munro KM, Adams JH. Acute ethylene glycol poisoning: report of a fatal case. Medicine, science, and the law. 1967;7:181-4.
33. Miller H, Barceloux DG, Krenzelok EP, Olson K, Watson W. American Academy of Clinical Toxicology Practice Guidelines on the Treatment of Ethylene Glycol Poisoning. Ad Hoc Committee. J Toxicol Clin Toxicol. 1999;37:537-60. Epub 1999/09/25.
34. Kraut JA, Mullins ME. Toxic alcohols. New England Journal of Medicine. 2018;378:270-80.
35. McMartin K, Jacobsen D, Hovda KE. Antidotes for poisoning by alcohols that form toxic metabolites. Br J Clin Pharmacol. 2016;81:505-15.
36. Lao YE, Vartdal T, Froeyshov S, Latimer B, Kvaerner C, Mataric M, Holm P, Foreid S, Jacobsen D, McMartin K, Hovda KE. Fomepizole dosing during continuous renal replacement therapy - an observational study. Clin Toxicol (Phila). 2021:1-7. Epub 2021/09/30.
